# Circulating and intrahepatic antiviral B cells are defective in hepatitis B

**DOI:** 10.1172/JCI121960

**Published:** 2018-08-09

**Authors:** Alice R. Burton, Laura J. Pallett, Laura E. McCoy, Kornelija Suveizdyte, Oliver E. Amin, Leo Swadling, Elena Alberts, Brian R. Davidson, Patrick T.F. Kennedy, Upkar S. Gill, Claudia Mauri, Paul A. Blair, Nadege Pelletier, Mala K. Maini

**Affiliations:** 1 Division of Infection and Immunity, Institute of Immunity and Transplantation, and; 2 Department of Surgery, University College London, London, United Kingdom.; 3 Centre for Immunobiology, Barts and the London, London, United Kingdom.; 4 Division of Medicine, University College London, London, United Kingdom.; 5 Roche Innovation Center, Basel, Switzerland.

**Keywords:** Immunology, Infectious disease, B cells, Hepatitis, Tolerance

## Abstract

B cells are increasingly recognized as playing an important role in the ongoing control of hepatitis B virus (HBV). The development of antibodies against the viral surface antigen (HBV surface antigen [HBsAgs]) constitutes the hallmark of resolution of acute infection and is a therapeutic goal for functional cure of chronic HBV (CHB). We characterized B cells directly ex vivo from the blood and liver of patients with CHB to investigate constraints on their antiviral potential. Unexpectedly, we found that HBsAg-specific B cells persisted in the blood and liver of many patients with CHB and were enriched for T-bet, a signature of antiviral potential in B cells. However, purified, differentiated HBsAg-specific B cells from patients with CHB had defective antibody production, consistent with undetectable anti-HBs antibodies in vivo. HBsAg-specific and global B cells had an accumulation of CD21^–^CD27^–^ atypical memory B cells (atMBC) with high expression of inhibitory receptors, including PD-1. These atMBC demonstrated altered signaling, homing, differentiation into antibody-producing cells, survival, and antiviral/proinflammatory cytokine production that could be partially rescued by PD-1 blockade. Analysis of B cells within healthy and HBV-infected livers implicated the combination of this tolerogenic niche and HBV infection in driving PD-1^hi^atMBC and impairing B cell immunity.

## Graphical abstract

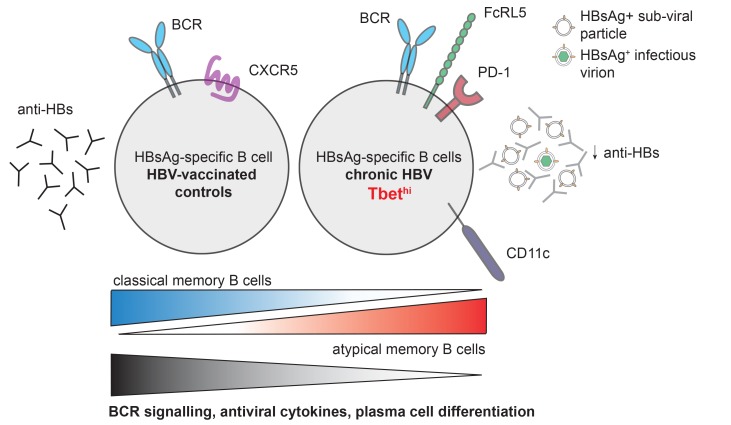

## Introduction

The humoral immune response is well recognized as playing an essential role in protection against pathogens. However, accumulating data implicate B cells and Abs, not only in protection against new infections, but also in their ongoing control (1–3). How their contribution may fail in chronic viral infections remains poorly understood; while much progress has been made in identifying targets underpinning T cell exhaustion in the setting of persistent antigenic stimulation, analogous defects in B cell responses are less well studied. A detailed understanding of the constraints on pathogen-specific B cell responses will identify new therapeutic targets for harnessing this arm of the immune response.

The prototypic humoral immune response is regarded as the differentiation of pathogen-specific memory B cells (MBC) into plasma cells capable of producing Abs that neutralize infectivity. However, other Ab effector functions often mediate additional important antiviral effects, using their capacity to harness innate and adaptive cellular responses. In HIV infection, Abs that are potently neutralizing have been shown to rely heavily on Fc receptor–binding activity for their efficacy (4). The Fc region of Abs allows them to leverage Ab-dependent cellular cytotoxicity (ADCC) or phagocytosis (ADCP) through binding to Fc receptors on NK cells or phagocytes, respectively, thereby promoting elimination of infected cells (5). In addition, by binding complement or forming immune complexes, Abs can promote complement-dependent cytotoxicity or antigen presentation to T cells, respectively (5–7). Beyond Abs, B cells are increasingly recognized as having additional important antiviral roles, including antigen presentation (7, 8) and the production of cytokines (9). Thus, it is crucial to consider the full spectrum of diverse antiviral roles of B cells that could be disabled in the setting of persistent viral infections.

The hepatotropic hepatitis B virus (HBV) can establish a chronic infection of a type that continues to kill more than 700,000 people every year and is therefore a major focus for the development of new therapeutic approaches (10). The efficacy of Abs directed against a component of the HBV envelope (HBV surface antigen [HBsAg]) in protection against de novo infection is well established: they are used therapeutically to limit maternofetal transmission or infection of liver transplants and constitute the basis of the preventative vaccine (11). Following successful resolution of acute HBV, evidenced by the development of anti-HBs Abs, residual intrahepatic HBV episomal DNA is successfully maintained under long-term immune control (10). Although the development of anti-HBs Abs is the hallmark of natural resolution of HBV, they have long been regarded as important in the prevention of reinfection rather than in ongoing immune control. However, an indispensable contribution of MBC to the control of residual infection has now been revealed by the observation that in vivo depletion of CD20-expressing cells by rituximab can drive viral rebound in some cases with serological evidence of prior HBV infection (anti-HBc ± anti-HBs) (12–14). Rituximab can precipitate HBV reactivation when used for B cell lymphomas, renal transplantation, or autoimmune diseases (12, 13), and the risk is increased in cases lacking preexisting anti-HBs Abs (14). Of note, rituximab does not deplete terminally differentiated plasma cells that typically downregulate CD20, suggesting a requirement for MBC for the maintenance of HBV control in these cases.

Despite this evidence for the importance of B cells in HBV control, little is known about the features and potential roles of B cell immunity once chronic HBV (CHB) infection is established. In this setting, subjects have Abs to HBV core antigen (HBcAg), a T cell–independent antigen that elicits a robust Ab response in all exposed individuals (15). However, these chronic carriers do not have detectable Abs to HBsAg in their sera (11). This implies that either concentrations of anti-HBs Abs synthesized are very low or that they are insufficient for overcoming absorption by the large quantities of HBsAg present in CHB. HBsAg is released as subviral particles in several-fold excess to full virions, which has been postulated as serving as an immune evasion mechanism, subverting any anti-HBs Abs produced and forming immune complexes in the circulation (16–18). An inherent deficiency of B cells able to synthesize anti-HBs Abs was first suggested as being a feature of CHB more than 30 years ago (19, 20). One potential mechanism for their depletion was proposed to be the deletion of B cells crosspresenting HBsAg on MHC class I by cytotoxic T lymphocytes (7). However, the lack of techniques for identifying antigen-specific B cells directly ex vivo has precluded determination of whether HBsAg-specific B cells are numerically depleted and/or functionally inhibited/defective. Moreover, other antiviral functions of B cells have not been comprehensively determined in CHB, nor have the characteristics of intrahepatic B cells been analyzed. Prior study of the peripheral global B cell compartment has shown impaired proliferation and increased expression of CXCR3 (21), while a regulatory population of B cells producing IL-10 has been shown to be expanded in flares of CHB disease activity (22). However, B cell production of IL-6 and TNF-α, cytokines with potent anti-HBV capacity (23–27), has not previously been assessed.

Here, we applied a fluorescent bait reagent allowing flow cytometric quantitation of HBsAg-specific B cells to reveal that they are maintained in many subjects with CHB and not reduced in frequency compared with vaccinated or naturally resolved controls. Using this method, we carried out direct ex vivo phenotypic profiling in a cohort of 84 patients with CHB to probe for mechanisms restraining their functionality. We found that a substantial proportion of HBsAg-specific B cells have the phenotype of atypical MBC (atMBC, CD21^–^CD27^–^, also known as tissue-like MBC or aged B cells), a functionally defective subset recently found to accumulate in settings of repetitive pathogen exposure (reviewed in refs. 28, 29). atMBC are also expanded within the peripheral global B cell compartment in CHB and, to a greater extent, in the HBV-infected liver of these individuals. We observe that the expanded atMBC in CHB have defective signaling, survival, antiviral cytokine production, and differentiation into Ab-producing cells. By defining their inhibitory receptor expression, we identify new targets for immunotherapeutic boosting of B cell immunity.

## Results

### HBsAg-specific B cells persist in CHB with impaired potential to produce Abs.

**Figure 1:**
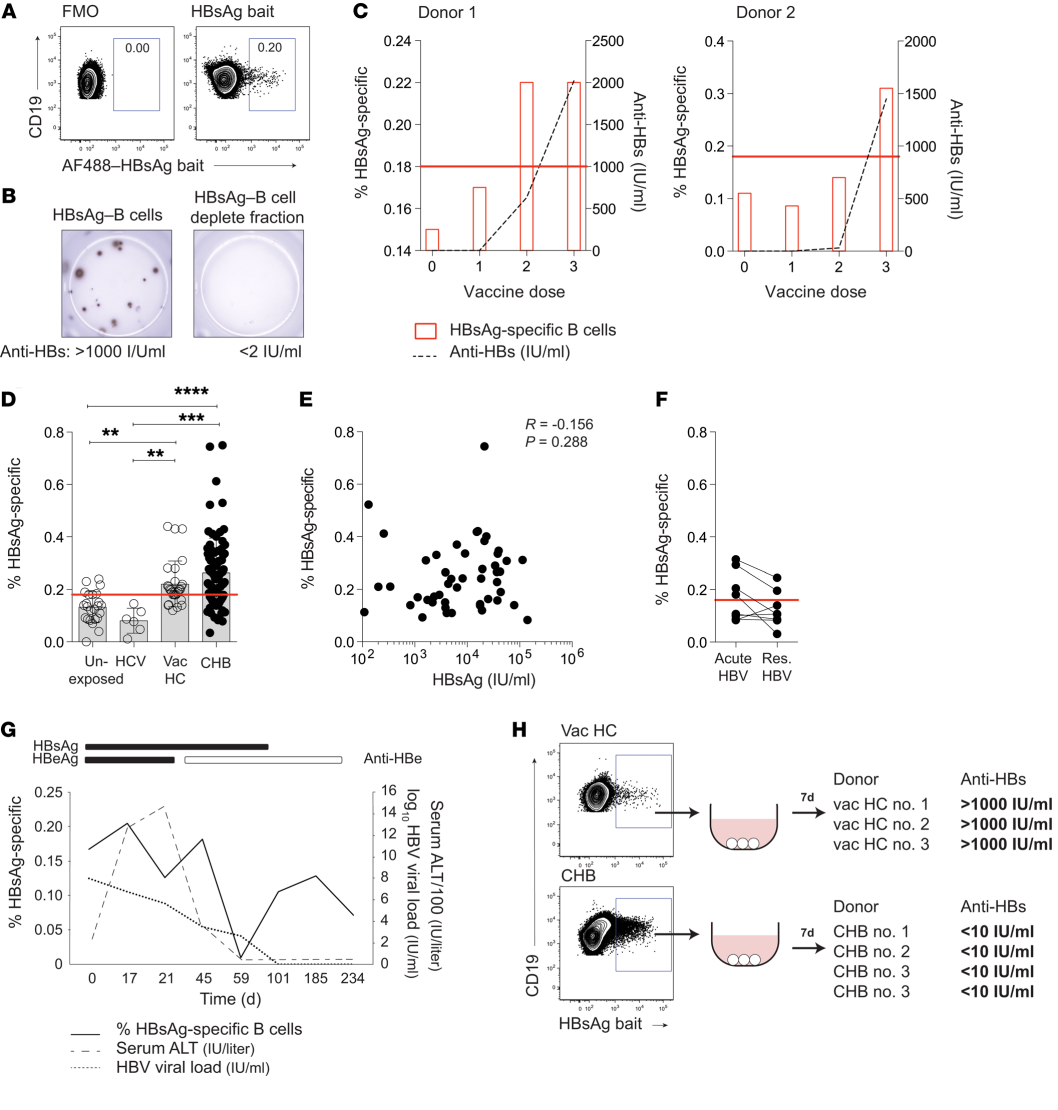
B cells specific for HBV surface antigen persist in chronic infection. (**A**) Representative staining: HBsAg-specific B cells in a vaccinated HC identified using an AF488–HBsAg bait, compared with FMO. (**B**) Representative ELISpot well image: anti-HBs–secreting B cells in HBsAg bait sorted– and HBsAg bait–depleted cells from HBV-vaccinated HC (representative of *n* = 3). anti-HBs measured in supernatant by ELISA (IU/ml). (**C**) HBsAg-specific B cells (red bars; % of total CD19^+^CD20^+^) across the course of HBV vaccination in 2 healthy donors. Samples taken 2 weeks prior to first dose and 7 days after each dose (given 1 and 6 months after the initial dose). Dashed line represents serum anti-HBs titer (IU/ml) determined by ELISA. Red line delineates threshold level of 0.18 based on mean + SD of unexposed controls. (**D**) Frequency of HBsAg-specific B cells in unexposed HC (*n* = 24), HBV-HCV^+^ patients (*n* = 6), HBV-vaccinated HC (vac HC; *n* = 29), and patients with CHB (*n* = 84) identified using AF488–HBsAg bait staining. Red line delineates threshold of detection, as above. (**E**) Frequency of HBsAg-specific B cells plotted against HBsAg titer (IU/ml; *n* = 48). (**F**) Cross-sectional analysis showing the frequency of HBsAg-specific B cells at HBV-acute and HBV-resolved (res.) time points (*n* = 8). (**G**) Longitudinal analysis of HBsAg-specific B cells during acute-resolving infection. Frequencies plotted relative to viral load (dashed line; IU/ml), serum ALT (dotted line; IU/liter), and serological status (indicated by bars). (**H**) anti-HBs in supernatants from stimulated FACS-sorted HBsAg-specific B cells (*n* = 3 HBV-vaccinated HC; *n* = 4 patients with CHB). Number of cells ranged from 5 × 10^3^ to 1.2 × 10^4^ for HBV-vaccinated HC and 5 × 10^3^ to 1.7 × 10^4^ in patients with CHB. Representative plot for HBV-vaccinated HC is also shown in Supplemental Figure 1A. Error bars indicate mean ± SEM. *P* values were determined by Kruskal-Wallis test (ANOVA) with Dunn’s post hoc test for pairwise multiple comparisons (**D**), Spearman’s rank correlation (**E**); and Wilcoxon’s paired *t* test (**F**). **P < 0.005; ***P < 0.001; ****P < 0.0001.

To investigate whether B cells specific for HBsAg circulate in chronic infection, we first tested a sensitive method for their direct ex vivo detection. Using a fluorescently labeled HBsAg bait, we were able to stain a population of antigen-specific B cells (CD45^+^CD3^–^CD19^+^CD20^+^) within peripheral blood mononuclear cells (PBMC) from an HBsAg-vaccinated donor (ENGERIX-B), allowing quantitation by flow cytometry (Figure 1A, gating strategy; Supplemental Figure 1A; supplemental material available online with this article; https://doi.org/10.1172/JCI121960DS1). The frequency of circulating HBsAg-specific B cells determined by this method was within the range for vaccinated donors estimated previously using a 2-step enrichment staining protocol (30). A threshold for a positive response was set using the mean + 1 standard deviation of the background staining seen in a cohort of unexposed controls (0.18% of B cells, Supplemental Figure 1B). This compromise cutoff left 4 of 24 unexposed donor stains just above the threshold, implying that responses at this threshold frequency must be interpreted with caution. To validate the reagent, we sorted the HBsAg bait–stained and bait–negative B cell fractions from a vaccinated donor and assessed their functionality after culture using ELISpot and ELISA. Cells selected using the HBsAg-specific bait differentiated into HBsAg-specific plasma cells detectable by ELISpot and produced more than 1,000 IU/ml of anti-HBs Ab by ELISA (Figure 1B). In contrast, cells from the bait-negative fraction were devoid of HBsAg-reactive responses, as determined by ELISpot, and produced no detectable anti-HBs Ab, as shown by ELISA (Figure 1B).

To further validate the specificity and sensitivity of the HBsAg bait, we used it to stain peripheral B cells from healthy donors sampled repeatedly during the course of preventative HBV vaccination (ENGERIX-B, containing recombinant HBsAg adsorbed on aluminium hydroxide). Detection of HBsAg-specific B cells above the background threshold of staining coincided with the development of a detectable anti-HBs Ab response in sera from 2 vaccinated donors (Figure 1C). Two donors who only received the first 2 doses of the vaccine failed to develop a detectable Ab response, as shown by ELISA, or an HBsAg-specific B cell response above the threshold (Supplemental Figure 1C).

Having validated the specificity of the HBsAg bait, we then used it to test for circulating HBsAg-specific B cells in a cohort of 84 subjects with CHB. Despite their lack of detectable serum anti-HBs Abs, we were able to detect HBsAg bait–staining B cells above the background threshold in 68% of the cohort at frequencies comparable to those of a cohort previously vaccinated with HBsAg (Figure 1D). Both subjects with CHB and vaccinees had significantly higher frequencies of HBsAg bait–staining B cells than unexposed controls or patients infected with HCV (Figure 1D). The frequency of HBsAg-specific B cells showed no relationship with circulating antigen load in vivo (serum HBsAg concentration, Figure 1E), HBV DNA, alanine transaminase (ALT), or clinical disease phase (Supplemental Figure 1, D–F). HBsAg-specific B cells were also detectable in some patients sampled during acute HBV, but were again at very low frequencies and showed a tendency to decrease rather than increase in the circulation when these donors were resampled around the time of HBsAg clearance (Figure 1F and Supplemental Figure 2, A and B). Temporal analysis through the course of acute-resolving HBV showed no consistent relationship with viral load, serology, or liver inflammation (Figure 1G and Supplemental Figure 2, A and B).

Next, we investigated whether HBsAg-specific B cells detected in CHB were capable of differentiating into Ab-producing cells. HBsAg-specific B cells FACS sorted from donors with CHB and cultured to promote differentiation into plasma cells failed to produce detectable levels of anti-HBs IgG (<10 IU/ml). In striking contrast, an equivalent number of bait-sorted HBsAg-specific B cells from vaccinated healthy controls (HC) differentiated in vitro to produce robust levels of anti-HBs (>1000 IU/ml) (Figure 1H). Thus, HBsAg-specific B cells were detectable directly ex vivo in CHB, but showed reduced capacity to produce anti-HBs Abs upon in vitro differentiation, consistent with the insufficient Ab production characteristic of this stage of infection.

### atMBC are expanded in CHB and enriched in the HBsAg-specific fraction.

To investigate this defect in Ab-producing potential of HBsAg-specific B cells circulating in CHB, we first dissected their composition according to well-described MBC subsets. To do this, we took advantage of the capacity to stain HBsAg-specific B cells directly ex vivo*,* allowing characterization by surface phenotype. Among antigen-experienced B cells, conventional MBC are characterized by the expression of CD27. They can be subdivided into classical MBC (cMBC), coexpressing CD27 and CD21 (component of the B cell receptor [BCR]), and activated MBC (actMBC), which retain CD27 but have downregulated expression of CD21. An additional subset, atMBC, lack expression of both CD27 and CD21 (28).

**Figure 2:**
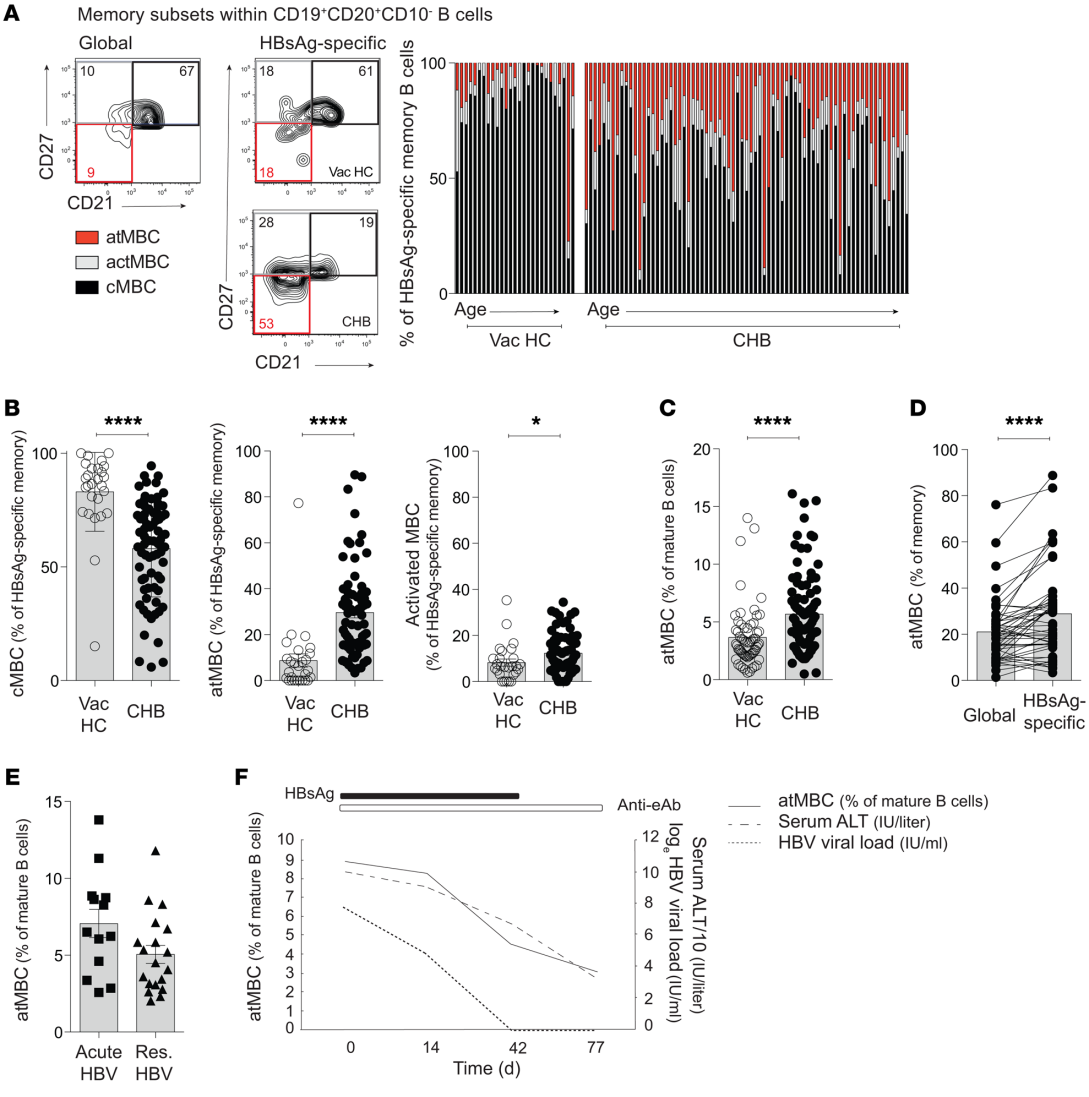
atMBC are expanded in HBV infection and enriched in the HBsAg-specific compartment. (**A**) Representative staining and cumulative data: HBsAg-specific MBC subsets (atMBC CD27^–^CD21^–^; actMBC CD27^+^CD21^–^, cMBC CD27^+^CD21^+^; gated on CD45^+^CD19^+^CD3^–^CD20^+^CD10^–^) in HBV-vaccinated HC (*n* = 27) and patients with CHB (*n* = 73). Each bar represents an individual. Individuals are ordered by increasing age (range: HBV-vaccinated HC = 21–89 years; CHB = 23–71 years). (**B**) Summary plots comparing the frequencies of HBsAg-specific MBC subsets between HBV-vaccinated HC (*n* = 27) and patients with CHB (*n* = 73). (**C**) Frequency of atMBC in the global B cell compartment in HBV-vaccinated HC (*n* = 61) and patients with CHB (*n* = 96). (**D**) Frequency of cells with an atMBC phenotype in the global compared with HBsAg-specific compartment (*n* = 49 patients with CHB). (**E**) Cross-sectional analysis of global atMBC in HBV-acute (*n* = 13) and HBV-resolved (*n* = 20) HBV infection (**F**) Longitudinal analysis of atMBC during acute-resolving infection. Frequencies plotted relative to viral load (dashed line; IU/ml), serum ALT (dotted line; IU/liter), and serological status (indicated by bars). Error bars indicate mean ± SEM. *P* values were determined by Mann-Whitney *t* test (**B**, **C**, and **E**) and Wilcoxon’s paired *t* test (**D**). *P < 0.05; ****P < 0.0001.

Using gating on CD10^–^CD19^+^CD20^+^ B cells (to exclude any contribution to the CD21^lo/–^ pool from immature transitional B cells or plasma cells), we compared the proportion of each subset as a proportion of memory HBsAg-specific B cells in healthy vaccinated donors and subjects with CHB (Figure 2A). Cells with a cMBC phenotype were the principal component of HBsAg-specific B cells in healthy vaccinated controls (Figure 2, A and B), consistent with the generation of a protective Ab response. However, in subjects with CHB, the HBsAg-specific cMBC population (CD27^+^CD21^+^) was contracted and partially replaced by B cells with an atMBC phenotype (CD27^–^CD21^–^) alongside a smaller expansion of CD27^+^CD21^–^ actMBC (Figure 2, A and B). In examining the whole cohort, we found that atMBC accounted for a mean of 30% (maximum 90%) of HBsAg-specific B cells in CHB, a 2.6-fold increase compared with vaccinated controls (Figure 2, A and B). The proportion of atMBC within HBsAg-specific B cells was not associated with age within these cohorts (Figure 2A) and only showed a weak inverse correlation with HBsAg load (out of all clinical parameters examined, Supplemental Figure 3, A–D).

We next questioned whether the expansion of atMBC was a generalized feature of CHB or was restricted to the HBsAg-specific compartment. We found that atMBC were also significantly increased within the global B cell pool in 96 donors with CHB compared with 61 uninfected controls (Figure 2C), although the enrichment was notably more striking within the HBsAg-specific fraction of MBC (Figure 2D). Again, the expansion of atMBC did not clearly correlate with any clinical parameters (Supplemental Figure 3, E–H), only showing a trend to be reduced in resolved compared with acute infection (Figure 2E). To investigate this in more detail, we took advantage of longitudinal samples from 3 patients taken during the course of acute-resolving HBV, revealing a tendency for atMBC to decrease following ALT flare (Figure 2F and Supplemental Figure 3I).

### atMBC in CHB are T-bet^hi^ with a homing profile favoring inflamed nonlymphoid tissues.

**Figure 3:**
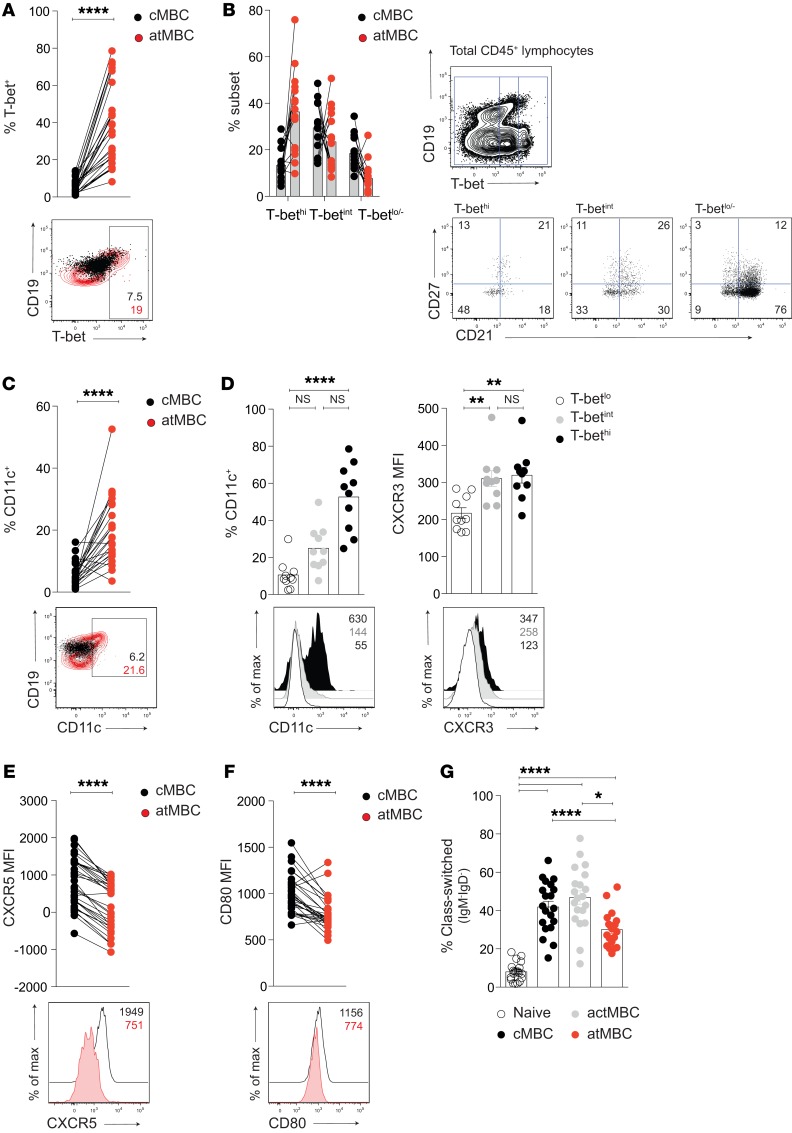
atMBC in CHB have altered T-bet expression and homing profiles. Representative examples and cumulative data: expression of (**A**) T-bet on global atMBC and cMBC (%; *n* = 30) and (**B**) percentage of MBC subsets within T-bet^hi^, T-bet^int^, or T-bet^lo^ fractions (pregated on CD20^+^CD19^+^CD10^–^; *n* = 15). Gates were drawn on total CD45^+^ lymphocytes, as shown. (**C**) Expression of CD11c (%; *n* = 24) on global atMBC and cMBC in patients with CHB. (**D**) Expression of CD11c (%; *n* = 10) and CXCR3 (mean fluorescence intensity [MFI]; *n* = 10) on T-bet^hi^ (black), T-bet^int^ (gray), or T-bet^lo^ (white) atMBC. (**E** and **F**) Expression of (**E**) CXCR5 (MFI; *n* = 33) and (**F**) CD80 (MFI; *n* = 30) on global atMBC and cMBC in patients with CHB. (**G**) Frequency of class-switched cells (IgM^–^IgD^–^) as a percentage of naive, cMBC, and atMBC (*n* = 39 patients with CHB). Error bars indicate mean ± SEM. *P* values were determined by Wilcoxon’s paired *t* test (**A**, **C**, **E**, and **F**) and Kruskal-Wallis test (ANOVA) with Dunn’s post hoc test for pairwise multiple comparisons (**D** and **G**). *P < 0.05; **P < 0.005; ****P < 0.0001.

To understand more about the features of the atMBC expanding in CHB and accumulating preferentially in the HBsAg-specific compartment, we probed for hallmarks of this dysfunctional population. The transcription factor T-bet has been shown to be induced in CD21^–^ B cells during murine and human viral infections, associated with the inflammatory homing markers CD11c and CXCR3 (29). We found that T-bet expression was increased in atMBC in CHB (particularly within the CD19^hi^ fraction) compared with their cMBC counterparts; consistent with this, the majority of B cells within the total T-bet^hi^ fraction had an atMBC phenotype (Figure 3, A and B).

atMBC in CHB expressed higher levels of the inflammatory homing marker CD11c compared with cMBC (Figure 3C). Expression of CD11c and the liver-homing chemokine CXCR3 was enriched within T-bet^int^ and T-bet^hi^ B cells (Figure 3D), as previously described (29, 31, 32). In contrast, the lymph node–homing chemokine CXCR5 was expressed on cMBC, but not on atMBC (Figure 3E). Congruent with this, atMBC had lower expression of the activation/costimulation marker CD80 than cMBC (Figure 3F), and the proportion that had undergone class switching was intermediate between naive and cMBC (Figure 3G). Combined, these data indicate that the profile of atMBC in CHB does not support homing to secondary lymphoid organs for the productive T cell interactions required for protective Ab production (33), but instead may favor migration to inflamed tissues, such as the HBV-infected liver.

### atMBC in CHB express multiple inhibitory receptors.

**Figure 4:**
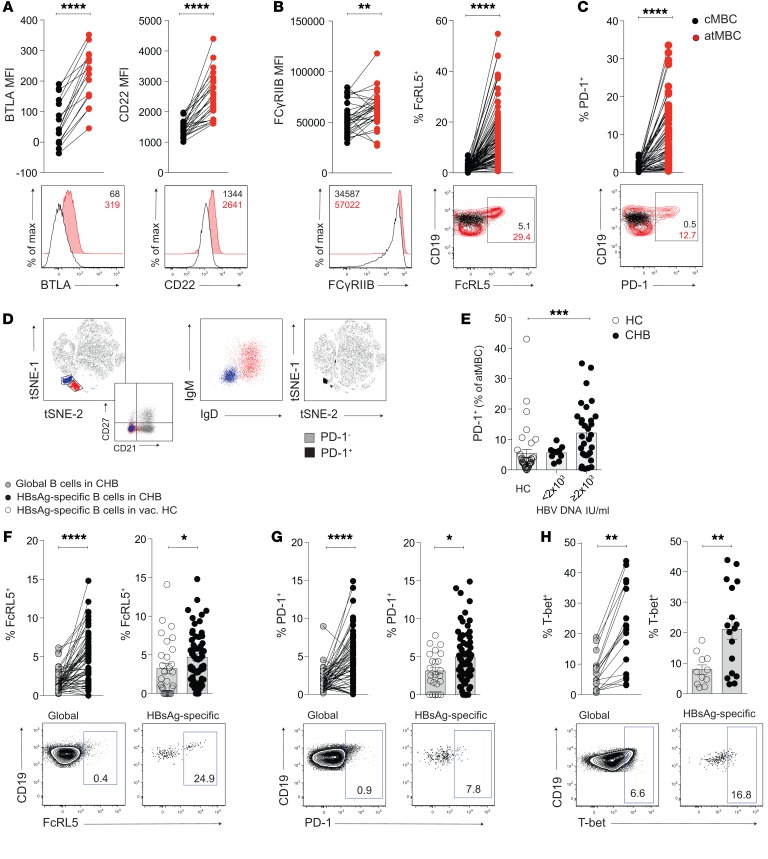
atMBC express higher levels of inhibitory receptors. (**A**–**C**) Representative examples and cumulative data: expression of (**A**) BTLA (MFI; *n* = 16) and CD22 (MFI; *n* = 26); (**B**) FcyRII1B (MFI; *n* = 30) and FcRL5 (%; *n* = 83); and (**C**) PD-1 (%; *n* = 55) on atMBC and cMBC in patients with CHB. (**D**) Dimension reduction analysis visualized using tSNE identifying discrete populations of atMBC based on the expression profile CD21^–^CD27^–^ FcRL5^+^. PD-1 expression on B cells was concentrated within IgM^–^IgD^–^ atMBC (purple cluster). tSNE analysis was performed on the expression data for the markers BAFF-R, IgD, CD21, CD80, CD10, CD11c, CD27, FcRL5, CD20, IgM, PD-1, CD38, and CD24 as measured by flow cytometry on CD19^+^ events concatenated from patients with CHB (*n* = 8) and HBV-vaccinated HC (*n* = 8). (**E**) Frequencies of PD-1^+^ atMBC stratified by viral load (IU/ml) (*n* = 10 with HBV DNA <2 × 10^3^; *n* = 31 with HBV DNA ≥2 × 10^3^) and compared with HC (HC; *n* = 37). (**F**–**H**) Representative examples and cumulative data: paired analysis of marker expression on HBsAg-specific B cells (black) compared with global B cells (gray) from within the same patient with CHB and comparison of HBsAg-specific B cells in patients with CHB and vaccinated HC (white). Expression levels of (**F**) FcRL5 (%; *n* = 60 patients with CHB; *n* = 29 HBV-vaccinated HC), (**G**) PD-1 (%; *n* = 66 patients with CHB; *n* = 23 HBV-vaccinated HC), and (**H**) T-bet (%; *n* = 17 patients with CHB; *n* = 11 HBV-vaccinated HC). Error bars indicate mean ± SEM. *P* values were determined by Wilcoxon’s paired *t* test (**A**–**C**; **F**–**H**), Kruskal-Wallis test (ANOVA) with Dunn’s post hoc test for pairwise multiple comparisons (**E**), and Mann-Whitney *t* test for unpaired data (**F**–**H**). *P < 0.05; **P < 0.005; ***P < 0.001; ****P < 0.0001.

In addition to the impaired signals attributable to downregulation of CD27 and CD21, atMBC can be constrained by inhibitory receptors. Such inhibitory receptors limit BCR signaling (28, 34–36) in a manner analogous to the well-described function of checkpoints on exhausted T cells. Known inhibitory receptors include B and T lymphocyte attenuator (BTLA) and CD22, which were both increased on atMBC compared with cMBC in CHB (Figure 4A). Two further inhibitory receptors, Fc_y_RIIB and FcRL5, which bind to the Fc portion of Abs (37–39), were also upregulated on CHB atMBC (Figure 4B). The expression of FcRL5, in particular, was strikingly increased compared with the minimal expression seen on cMBC (Figure 4B). Finally, the prototypic T cell checkpoint PD-1 (40) was also expressed on a subset of atMBC and barely expressed on cMBC (Figure 4C).

Dimension reduction analysis using t-distributed stochastic neighbor embedding (tSNE) showed that atMBC clustered as 2 discrete populations when defined by the expression of FcRL5 in addition to the CD27^–^CD21^–^ phenotype (Figure 4D). Global B cell PD-1 expression was concentrated in these regions, particularly the cluster negative for IgM (class-switched FcRL5^+^ B cells lacking CD21 and CD27), implicating it as a hallmark of antigen-experienced atMBC in CHB (Figure 4D). Although some atMBC in HC and low-level HBV carriers expressed PD-1, this proportion was more than doubled in HBV carriers with higher viral loads (>2,000 IU/ml, Figure 4E).

To further investigate the relevance of these changes to antiviral immunity, we next examined the inhibitory receptor profile of HBsAg-specific B cells in patients with CHB, identified directly ex vivo by bait staining. The expression of both FcRL5 and PD-1 was consistently enriched on HBsAg-specific B cells compared with their global B cell counterparts or with HBsAg-specific B cells in vaccinated controls (Figure 4, F and G). The other inhibitory receptors examined (BTLA, CD22, and Fc_y_RIIB) were enriched on HBsAg-specific compared with global B cells, but not compared with responses in vaccinees (Supplemental Figure 4, A–C). B cells expressing high levels of the transcription factor T-bet were increased more than 2-fold in the HBsAg-specific compared with the global B cell compartment in CHB or compared with HBsAg-specific B cells in uninfected vacinees (Figure 4H). In line with this, HBsAg-specific B cells also expressed more CD11c and less CXCR5 in CHB than controls (Supplemental Figure 4, D and E). In addition to being linked with atypical/aged MBC, T-bet has also been shown to be required for isotype switching and antiviral function in B cells (29, 31, 41–43). Thus PD-1^hi^T-bet^hi^HBsAg-specific B cells may represent key antiviral effectors in CHB that are driven toward functional exhaustion by repetitive antigenic stimulation.

### atMBC accumulating in CHB have impaired signaling and antiviral function, partially rescued by PD-1 blockade.

**Figure 5:**
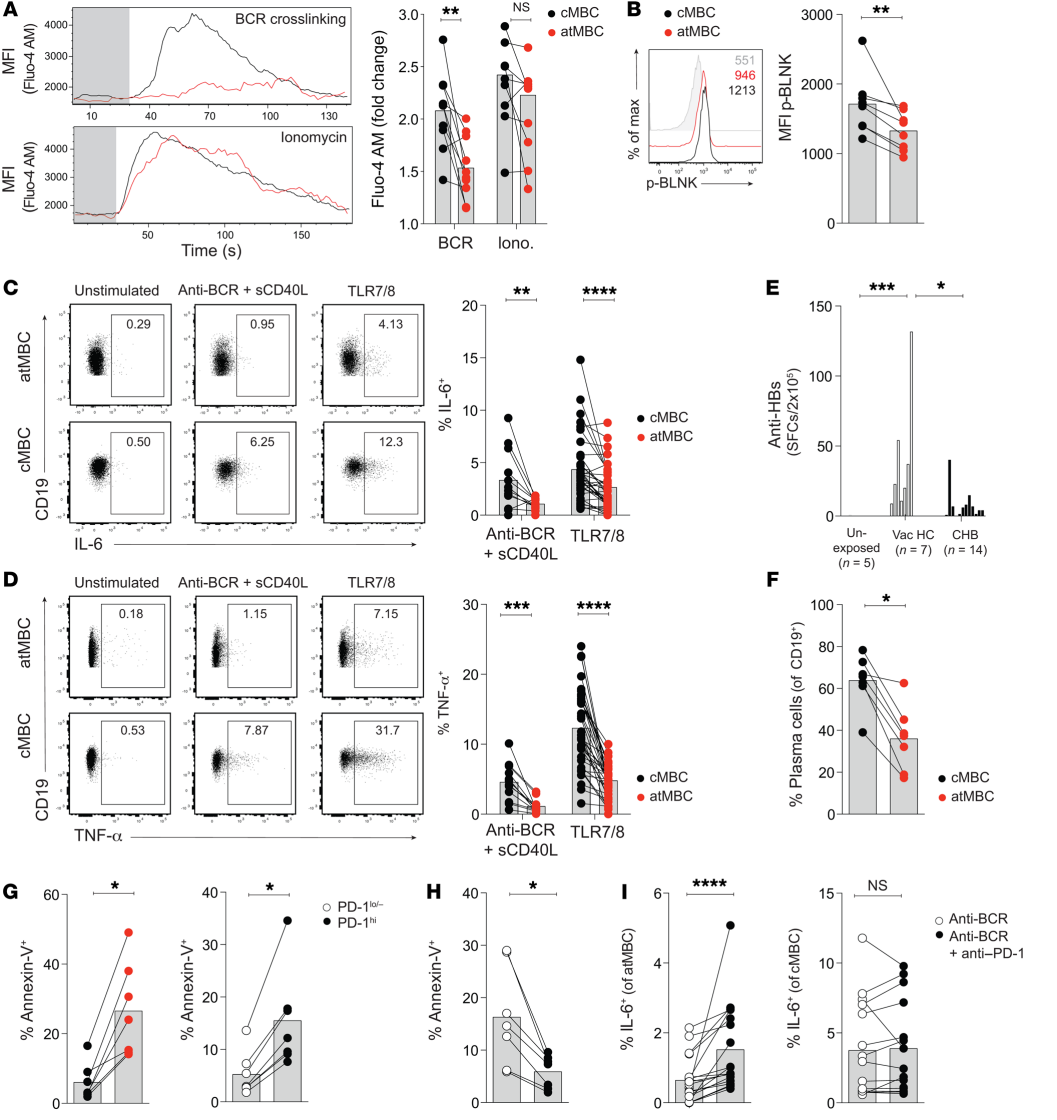
atMBC accumulating in CHB have impaired signaling and antiviral function. (**A**) Representative flow cytometric analysis of Ca^2+^ influx (Fluo-4 AM; median fluorescence intensity) over time (seconds) in purified B cells after stimulation with F(ab′)_2_-IgG/IgA/IgM (anti-BCR; 50 μg/ml) or ionomycin (iono.) (1 μg/ml) (*n* = 10 patients with CHB). Basal fluorescence prior to stimulation is shaded gray. Summary plot: difference in MFI upon stimulation in cMBC and atMBC compared with baseline. (**B**) Expression of phosphorylated-BLNK (MFI) in global B cells after crosslinking with F(ab′)_2_-IgG/IgA/IgM for 30 seconds (anti-BCR; *n* = 8). Background fluorescence from paired stimulated control is shown in gray. (**C**–**D**) Intracellular cytokine staining for (**C**) IL-6 and (**D**) TNF-α in atMBC and cMBC after stimulation with F(ab′)_2_-IgG/IgA/IgM and CD40L (anti-BCR; soluble CD40L [sCD40L]; *n* = 10 patients with CHB) or R848 (resiquimod; TLR7/8 agonist; 1 μg/ml; *n* = 35 patients with CHB) for 24 hours. Frequencies are presented minus paired unstimulated control. (**E**) Anti-HBs–secreting B cells in unexposed controls (*n* = 5), vaccinated HC (*n* = 7), and patients with CHB (*n* = 14), determined by ELISpot. SFC, spot-forming cells. (**F**) atMBC were FACS sorted (*n* = 7 HBV-vaccinated HC) and differentiated into plasma cells alongside a matched number of cMBC. Graph shows proportion of cells acquiring a plasma cell phenotype (CD45^+^CD19^+^CD3^–^IgD^–^CD38^hi^CD20^–^CD27^+^CD138^+^), as determined by flow cytometry. (**G**) Ex vivo staining for annexin V on purified B cells, stratified by subset and by PD-1 expression, after stimulation with F(ab′)_2_-IgG and -IgM (1 μg/ml) and CD40L (0.5 μg/ml) for 7 days (*n* = 7). (**H**) Annexin V expression on B cells stimulated ± anti–PD-1 mAb (10 μg/ml) for 7 days (*n* = 7). (**I**) Intracellular staining for IL-6 on atMBC and cMBC stimulated as in **G** and **H** for 24 hours (*n* = 18). Frequency is presented minus paired unstimulated control. Error bars indicate mean ± SEM. *P* values were determined by Wilcoxon’s paired *t* test (**A**–**D**, **F**–**I**); and Kruskal-Wallis test (ANOVA) with Dunn’s post hoc test for pairwise multiple comparisons (**E**). *P < 0.05; **P < 0.005; ***P < 0.001; ****P < 0.0001.

Next, we investigated whether the phenotypic changes observed in the expanded population of atMBC in CHB resulted in impaired functionality. To test responsiveness to antigen, we quantified calcium flux upon BCR engagement, a measure of proximal B cell signaling reflective of the capacity for subsequent differentiation and effector function (44). Engagement of the BCR induced robust calcium mobilization in cMBC; this change in calcium levels upon BCR stimulation was markedly reduced in atMBC (Figure 5A), as previously reported in subjects with malaria (44). In contrast, when stimulated with ionomycin, atMBC and cMBC demonstrated a comparable capacity to mobilize calcium, suggesting that the atMBC defect could be bypassed by a strong BCR-independent signal (Figure 5A). Phosphorylation of B cell linker protein (BLNK), an adaptor molecule important in coordinating BCR signaling, was also significantly diminished in atMBC compared with cMBC following BCR crosslinking (Figure 5B). Together, these data suggested that atMBC in CHB have attenuated BCR signaling, impairing their activation in response to antigen, but this reflects a “dampening” rather than complete unresponsivensss, suggesting the potential for rescue.

To test atMBC antiviral function, we first analyzed the capacity of atMBC to produce cytokines in patients with CHB. B cells are increasingly recognized as being an important source of a number of cytokines in viral infections and other settings (9, 22, 45), among which both TNF-α and IL-6 could have potent noncytolytic antiviral activity against HBV (23–27). The percentage of cells able to produce IL-6 or TNF-α upon crosslinking of the BCR in the presence of CD40 ligand (CD40L) was significantly less in atMBC compared with cMBC (Figure 5, C and D). Likewise, when stimulated with a TLR7/8 agonist (resiquimod, R848), atMBC were significantly impaired in their ability to produce both cytokines (Figure 5, C and D). These data suggest that atMBC in patients with CHB are functionally impaired, with reduced capacity to secrete important antiviral cytokines.

Another key role of MBC in pathogen immunity is their differentiation into Ab-secreting plasma cells. Initial examination of plasma cell formation by ELISpot showed that global B cells isolated from patients with CHB had defective differentiation to anti-HBs–secreting plasma cells compared with vaccinated controls (Figure 5E), as previously reported (46–48). We therefore investigated whether impaired differentiation into anti-HBs–producing cells in CHB was partially attributable to their enrichment of atMBC. Pure populations of atMBC and cMBC were FACS sorted and stimulated with HBsAg, a TLR9 agonist (CpG-B), and cytokines to compare their capacity to differentiate into plasma cells (IgD^–^CD38^hi^CD20^lo^CD138^+^CD27^+^). Comparison of a matched number of starting cells showed that significantly fewer atMBC differentiated into plasma cells compared with cMBC from the same donor (Figure 5F and Supplemental Figure 5). This suggests that impaired differentiation of atMBC into Ab-secreting cells may limit their production of anti-HBs.

During plasma cell differentiation, we noted reduced survival of those derived from atMBC compared with cMBC (Supplemental Figure 5). Therefore, to determine whether increased susceptibility to cell death might also be a factor limiting the differentiation of atMBC to plasma cells in vivo, we stained them after BCR stimulation for the apoptosis marker annexin V. There was an increased proportion of atMBC-expressing annexin V compared with cMBC, indicative of an increased propensity to apoptosis (Figure 5G). Higher annexin V was seen on PD-1^hi^ B cells (Figure 5G), in line with the known proapoptotic function of this molecule in B cells (49).

Since increased PD-1 was a prominent feature of atMBC and HBsAg-specific B cells in CHB and is a clinically applicable immunotherapeutic target (10), we tested the potential of PD-1 blockade to rescue B cell responses in CHB. B cells within PBMC were stimulated via their BCR and CD40 in the presence or absence of anti–PD-1 blocking Abs. PD-1 blockade was able to significantly reduce B cell apoptosis, marked by annexin V (Figure 5H). To determine whether PD-1 blockade could rescue the effector potential of B cells in addition to their survival, we examined changes in their production of the key antiviral cytokine IL-6. The proportion of atMBC able to produce IL-6 upon BCR crosslinking was significantly boosted in the presence of PD-1 blockade (Figure 5I). Consistent with their lower expression of PD-1, cMBC did not increase IL-6 production upon PD-1 blockade (Figure 5I).

Taken together, these data suggest that the reduced signaling and higher propensity to apoptosis observed in B cells in CHB that have downregulated CD21 and CD27 and upregulated inhibitors such as PD-1 impair their antiviral capacity by reducing production of IL-6 and TNF-α as well as limiting their differentiation into Ab-producing cells.

### Antigen-specific and PD-1^hi^ atMBC localize in the HBV-infected liver.

**Figure 6:**
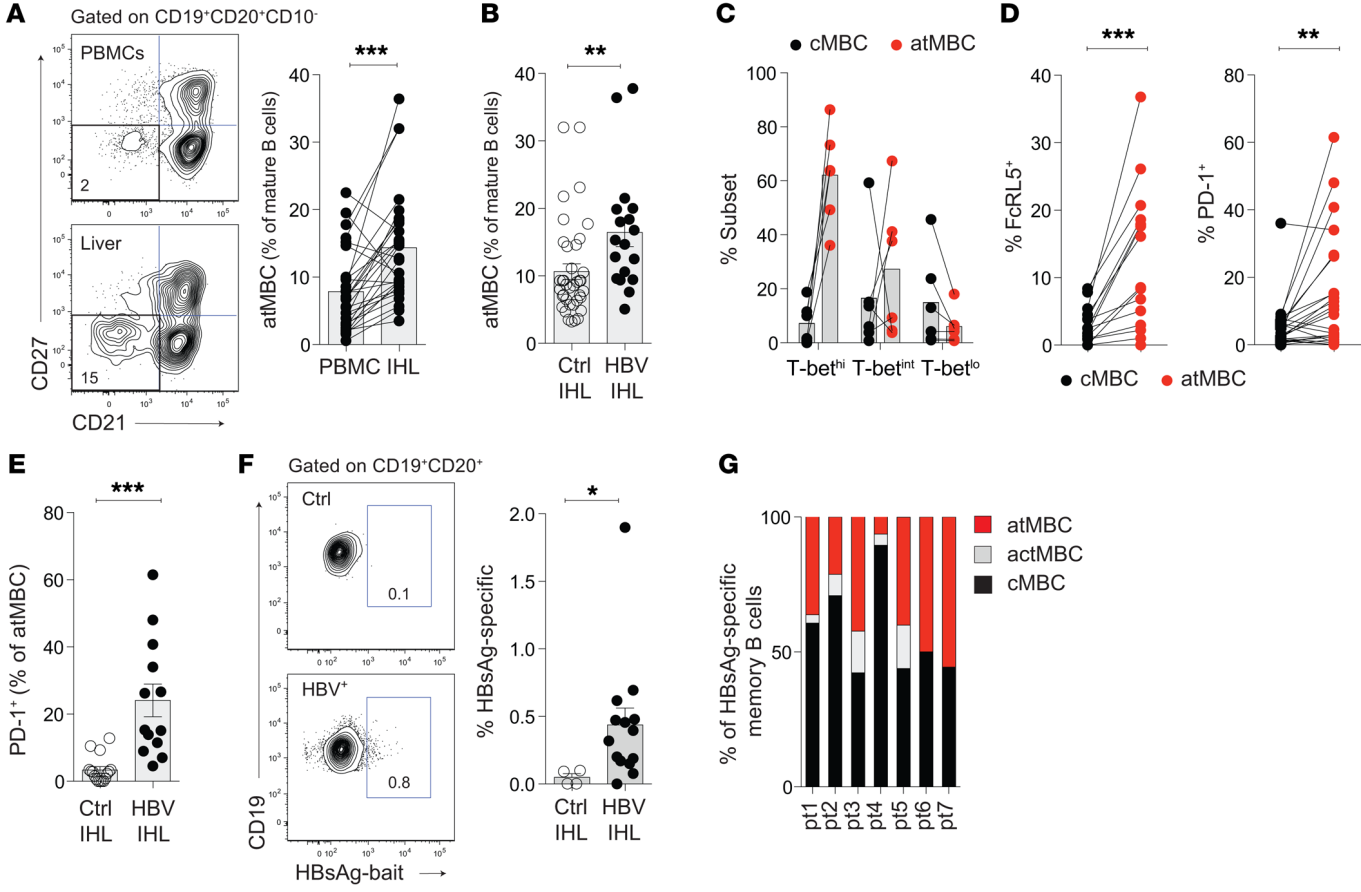
PD-1^hi^ atMBC preferentially localize in the HBV-infected liver. (**A**) Representative staining and summary plot: frequencies of atMBC in paired IHL and blood samples (PBMC) from patients with CHB (10 HBV^+^ liver biopsies; 5 HBV^+^ liver resections) and uninfected controls (12 margins from HBV^–^ colorectal metastases [CRC margins]; control IHL). (**B**) Frequencies of atMBC in control liver samples (22 CRC margins; 12 pretransplant perfusates; 6 biopsies from livers with nonviral hepatitis) and HBV-infected liver (10 HBV^+^ liver biopsies; 1 perfusate from HBsAg^+^ liver; 2 perfusates from HBV-resolved livers). Ctrl, control. (**C**) Percentage of intrahepatic B cells with atMBC or cMBC phenotype within global T-bet^hi^, T-bet^int^, or T-bet^lo^ populations (*n* = 2 CRC; 3 HBV^+^ tissue; 1 healthy perfusate). (**D**) Expression of FcRL5 (*n* = 4 HBV^+^ infected liver; 12 CRC margins) and PD-1 (*n* = 12 HBV^+^ liver; 13 CRC margins) on intrahepatic atMBC and cMBC. (**E**) Comparison of PD-1^+^ atMBC in uninfected liver (%; *n* = 15 CRC margins) and HBV^+^ liver (*n* = 13). (**F**) Representative staining and frequency of HBsAg-specific B cells (% of CD19^+^CD20^+^) in HBV^+^ liver samples (*n* = 14) compared with uninfected controls (*n* = 4 CRC margins). (**G**) Frequencies of HBsAg-specific MBC subsets in 7 individual HBV^+^ liver samples (patient [pt.] 1 through pt. 7). Error bars indicate mean ± SEM. *P* values were determined by Wilcoxon’s paired *t* test (**A** and **D**) and Mann-Whitney *t* test (**B**, **E**, and **F**). *P < 0.05; **P < 0.005; ***P < 0.001.

atMBC in CHB expressed homing receptors that would be expected to promote their accumulation in inflamed sites, such as the liver. Little is known about B cells in the liver, the site of HBV replication and inflammation, and a tolerogenic organ. We postulated that the microenvironment of the HBV-infected liver may contribute to the accumulation of exhausted atMBC in this infection. Comparison of 27 paired blood and liver tissue samples from patients with CHB or uninfected controls revealed that the increase in atMBC seen in the global B cell compartment in the periphery was even more exaggerated in the liver (Figure 6A). The frequency of atMBC was particularly enriched in HBV-infected compared with uninfected livers (healthy margins of metastases or nonviral hepatitis, Figure 6B), pointing to a combined effect of the liver milieu and the virus in expanding this subset.

As noted in the periphery, atMBC in the liver had increased expression of the transcription factor T-bet, accounting for the large majority of the T-bet^hi^ fraction within intrahepatic B cells (Figure 6C). Intrahepatic atMBC also had significantly higher expression of inhibitory receptors, such as FcRL5 and PD-1, than cMBC in the liver (Figure 6D). Upregulation of PD-1 was particularly striking in HBV-infected livers, where a maximum of 62% and a mean of 28% of atMBC were PD-1^+^, contrasting with a mean of only 3% in healthy liver (Figure 6E).

Finally, in a subset of samples from HBV-infected livers, we obtained sufficient cells to stain with the HBsAg-specific bait and investigate whether HBV-specific B cells can infiltrate the site of infection. HBsAg-specific B cells were detectable within lymphocytes isolated from all HBV-infected livers tested, whereas only background bait staining was seen among lymphocytes extracted from control HBV-negative liver samples (Figure 6F). As observed in the periphery, intrahepatic HBsAg-specific B cells were enriched for atMBC (Figure 6G).

These data show that atMBC preferentially accumulated in the liver niche and were further expanded in the presence of HBV infection, which drove upregulation of the inhibitory receptor PD-1. They demonstrate that virus-specific MBC can localize at the site of infection and pathology, allowing their phenotype and function to be shaped by the organ-specific milieu.

## Discussion

Accumulating clinical evidence supports an important role for B cells in the immune control of HBV, mandating study of their defects in persistent infection that affects an estimated 290 million people worldwide (50). The hallmark of naturally resolving HBV infection is the development of Abs directed against HBsAg, yet little is known about what limits their production in CHB. In this study, we show that HBsAg-specific B cells circulate in many patients with CHB, but have defective production of anti-HBs Abs. Congruent with this functional deficit, pathogen-specific B cells and, to a lesser extent, the global B cell compartment have an accumulation of atMBC enriched for coinhibitory receptors, including PD-1 and FcRL5. atMBC from patients with CHB show attenuated signaling and cytokine production in response to BCR triggering and a diminished ability to escape apoptosis and differentiate into Ab-producing plasma cells. In vitro PD-1 blockade with CD40L stimulation exemplifies the potential for therapeutic enhancement of B cell immunity in patients with CHB. Finally, we find that HBsAg-specific B cells can localize in the infected liver, where atMBC preferentially accumulate and upregulate PD-1.

atMBC have previously been identified in lymphoid tissue (51); we now find that they can also be sequestered in nonlymphoid organs, such as the liver. To our knowledge, this is also the first demonstration that human pathogen-specific MBC can localize within an infected organ. This finding has important implications for their roles at the site of viral replication and disease pathology. First, it implies that it may be possible to boost intrahepatic plasma cells capable of producing anti-HBs Abs with neutralizing or other antiviral capacity. Local production of anti-HBs Abs would facilitate their binding to intrahepatic virions to block infection of new hepatocytes or to HBsAg on the hepatocyte membrane to promote elimination through ADCC. Work using a hepatoma cell line has suggested that hepatocytes may also be able to take up anti-HBs Abs through FcRn receptors that could then directly exert intracellular antiviral effects (52). Furthermore, local production of antiviral cytokines by intrahepatic pathogen-specific B cells could optimize their delivery to infected hepatocytes. TNF-α, together with IFN-γ, plays a central role in the noncytolytic control of HBV replication and can degrade cccDNA (23, 26). Similarly, IL-6 has been shown to inhibit HBV at multiple steps of its life cycle, including downregulating the entry receptor (27) and inhibiting transcription and cccDNA acetylation (24, 25). IL-6 is also essential for maturation of activated B cells into immunoglobulin-producing cells (53), promoting T follicular helper cell (Tfh) generation for viral control in the late stages of infection (54). The reduced production of IL-6 and TNF-α that we documented by atMBC in CHB would therefore limit their direct antiviral activity and, combined with their reduced expression of CD80, could impair productive interactions with T cells. Future studies should investigate the potential for B cell–T cell crosstalk in the HBV-infected liver, since intrahepatic B cell follicles representing possible ectopic germinal centers (55) and Tfh cells (56) have been observed in HCV-infected livers.

Although intrahepatic HBV-specific B cells could favor local antiviral efficacy, the hostile liver environment would need to be overcome for their optimal functioning. Our finding of preferential accumulation of atMBC within the liver compared with the circulation suggests that the hepatic microenvironment may promote the development of this dysfunctional phenotype of MBC. This is consistent with previous findings pointing to the combination of BCR engagement, proinflammatory cytokines (IFN-γ), and TLR signals (TLR7/9), all features of the HBV-infected liver, as key factors driving T-bet and atMCB (29, 42, 57–60). B cells with downregulated CD21 and CD27 (atMBC) and upregulated PD-1, FcRL5, and CD11c expression were increased within the HBsAg-specific compartment in CHB compared with both their global B cell counterparts and with HBsAg-specific B cells in vaccinated controls. The enrichment of PD-1^hi^FcRL5^hi^ atMBC within the HBsAg-specific pool, as previously described for gp140-specific B cells in HIV, supports a central role for viral antigen driving their atypical phenotype through repetitive BCR engagement (28, 31). Although atMBC were also present in non–HBV-infected livers, they were more frequent in those infected with HBV and expressed more PD-1 in the latter group. These findings, from ex vivo examination of B cells freshly isolated from a large number of human liver samples, therefore, support a combined effect of the tolerogenic liver milieu and HBV viral antigen in driving the atypical and PD-1^hi^ phenotype. This is analogous to our recent observations of PD-1 on CD8^+^ T cells, which is also increased in the intrahepatic compared with circulating fraction, with expression further upregulated in HBV-infected compared with healthy livers (61).

We focused initially on the potential role of PD-1 in constraining atMBC, as it was enriched on HBsAg-specific and intrahepatic atMBC, and PD-1 blockade is already being tested in patients with CHB based on its capacity to boost antiviral T cells (10). Multidimensional analysis showed that PD-1 expression on B cells in CHB primarily clustered with the class-switched FcRL5^+^ atMBC fraction. High PD-1 expression on atMBC and on HBsAg-specific B cells was accompanied by increased intracellular levels of T-bet. T-bet has been suggested as defining a population of B cells with high antiviral potential (29, 31, 41–43), but also associates with the PD-1^hi^ atypical dysfunctional phenotype in CHB, as in malaria, HCV, and HIV (28, 29, 58, 62). These paradoxical findings are reminiscent of the complex role of PD-1 on T cells, associated with both activation and inhibition, limiting T cell function while also promoting long-term maintenance of immunosurveillance in the setting of chronic antigenic stimulation (40, 61, 63). Overall, they support T-bet^hi^PD-1^hi^HBsAg-specific B cells being a favorable target for releasing from inhibition, since they should be transcriptionally wired for antiviral efficacy. Consistent with this, we were able to enhance the capacity of atMBC from patients with CHB to produce the antiviral B cell differentiation-promoting cytokine IL-6 with short-term PD-1 blockade and CD40L stimulation. Although the majority of studies have focused on PD-1 in T cells, several reports have shown that this receptor can also inhibit B cell signaling, survival, and functionality (34–36, 49, 64, 65). Of particular note, in vivo PD-1 blockade in macaques with SIV was able to enhance MBC proliferation and production of SIV envelope–specific Abs in parallel with T cell reconstitution (64). Indirect effects of PD-1 blockade on B cells via boosting of Tfh cannot be excluded in vivo, although previous work in mice has suggested PD-1 blockade actually inhibits germinal center Tfh interactions (66). A direct effect of PD-1 blockade on B cells was supported by further work from SIV-infected macaques showing preferential depletion of PD-1–expressing MBC in vivo and the susceptibility of PD-1^hi^CD21^–^ actMBC to PD-L1–induced apoptosis in vitro (49). The latter finding is in keeping with our data showing that BCR-stimulated atMBC from patients with CHB underwent approximately 3-fold less apoptosis in the presence of PD-1 blockade.

Previous work has shown that B cell proliferation and cytokine production can be boosted by siRNA knockdown of PD-1 and a range of other inhibitory receptors expressed on atMBC from patients with HIV (34). The atMBC we identified in patients with CHB had significantly increased expression of a number of inhibitory receptors on their surface, as also noted in malaria (44); future studies will need to investigate their potential contribution to the impaired signaling and effector function we observed. Coligation of PD-1 and the BCR inhibits Ca^2+^ mobilization and tyrosine phosphorylation of several proximal BCR-signaling molecules, such as SYK (35), consistent with the impaired BCR-dependent calcium flux and reduced phosphorylation of BLNK we observed in atMBC in CHB. Analogous impairments have been observed as a result of ligation of the inhibitory receptor FcRL5, another hallmark of atMBC (44, 67), which we found to be markedly upregulated on atMBC and HBsAg-specific B cells in CHB. FcRL5 binds the Fc of intact IgG (38, 39), raising the possibility that circulating immune complexes crosslink FcRL5 to the BCR, further suppressing B cell activation in a way similar to that of the inhibitory Fc receptor FcγRIIB (37). Therefore, it would be of interest to examine whether circulating HBsAg/anti-HBs immune complexes, a well-recognized feature of CHB (16), further contribute to suppressing B cell function through the increased expression of FcRL5 and FcγRIIB.

Much remains to be understood regarding the complexity of B cell responses in HBV. The use of HBsAg baits for direct ex vivo quantification and phenotyping will facilitate further studies of B cell immunity in CHB, but has a number of caveats. As described for HLA/peptide multimer detection of HBV-specific T cells (68), HBsAg-specific B cells are often low frequency and can even approximate the background HBsAg bait stains seen in uninfected controls; however, background can be reduced by use of a dual-staining HBsAg bait (69). Furthermore, circulating HBsAg-specific B cell frequencies, for example, after resolution of acute HBV, are unlikely to be representative of MBC compartmentalized within lymphoid tissue. It is also conceivable that HBsAg-specific B cells may be further underestimated if HBsAg bound to their BCR in vivo prevents bait staining in vitro or if the Ab-binding site on sAg in the bait differs from that produced in vivo.

There is clearly heterogeneity between, and even within, the 2 atMBC subsets we identified. It will be important to investigate whether the IgM-expressing fraction of atMBC in CHB can synthesize Abs with pathogenic potential, as previously ascribed to intrahepatic IgM^+^anti-HBc Abs in HBV-associated acute liver failure (70). An accumulation of virus-specific B cells in the liver might also divert them away from the formation of long-lived plasma cells within the bone marrow, as suggested by the finding that rituximab, which spares the latter population, can result in reactivation of HBV (13). Another issue remaining to be addressed is that the inadequate Ab production by HBsAg-specific B cells cannot solely be attributed to the atMBC fraction expressing inhibitory receptors. There are likely additional, more generalized HBsAg-specific B cell defects related to the high-dose antigen and, in particular, to inadequate T cell help, consistent with a recent report of impaired HBsAg-specific Tfh function in HBV (71). Future studies could analyze HBsAg-specific B cells and Tfh in parallel to better define the influence of defective T cell help on B cell functionality. In addition, the availability of robust assays for quantitating and/or dissociating circulating HBsAg/Ab complexes would allow a better assessment of the influence of antigen load on HBsAg-specific B cell frequencies and Ab detection. However, the capacity of the large quantities of HBsAg in viral and subviral particles to drive B cell exhaustion and the extent to which subviral particles serve as a decoy for existing Abs (18) may soon be clarified by trials of novel therapies aiming to reduce HBsAg’s production or release (10).

In summary, our data show that HBV infection imprints major changes on the B cell compartments in both the periphery and liver. atMBC are expanded in global and HBsAg-specific B cells in CHB, are enriched in the liver, and express more inhibitory receptors such as PD-1 than their counterparts in uninfected controls. We show that many donors with CHB lacking detectable anti-HBs Ab associated with infection resolution do maintain MBC of this specificity in their circulation and liver, revealing the opportunity to boost functional antiviral responses. The transcriptional, phenotypic, signaling, and functional features of the atMBC in CHB suggest they contain effectors with antiviral potential that have become defective in the setting of chronic antigenic stimulation and inflammation. Our demonstration that their premature apoptosis and impaired antiviral function can be partially rescued by PD-1 blockade with CD40L stimulation paves the way for future attempts to optimize their therapeutic reconstitution. Along these lines, Salimzadeh et al. show that addition of PD-1 blockade to CD40L stimulation rescues anti-HBs Ab production from HBsAg-specific B cells sorted from 4 patients with CHB (69). PD-1 blockade is already being tested in the clinic for HBV and HBV-related HCC (10); it will be instructive to delineate whether there is any impact of this monotherapy on HBV-specific B cell frequencies and function in vivo.

Further studies will be needed to test additional stimuli bypassing the attenuated BCR pathway, compensating for inadequate Tfh help and/or blocking synergistic inhibitory receptors in order to optimize B cell recovery in CHB. The clinical application of such approaches will require caution because of the potential for B cells, like T cells, to drive tissue damage in tandem with antiviral immunity. Therapeutic blockade of inhibitory receptors is likely to preferentially expand the T-bet^hi^CD21^–^CD27^–^ subset of B cells, which is strongly implicated in autoimmunity (59). Pertinent to this concept, a recent report of melanoma patients treated with combination CTLA-4 and PD-1 blockade associated their risk of developing immune-related adverse events with the expansion of CD21^lo^ B cells with a nonlymphoid tissue–homing profile (72). In CHB, an alternative to B cell boosting is the direct administration of therapeutic monoclonal Ab infusions, which has already been shown to achieve short-term reductions in HBsAg and HBV DNA (73, 74). Recently, an Ab able to optimize ADCP and indirectly boost T cells resulted in more sustained effects in mouse models of HBV (73–75), underscoring the impact of effective humoral immunity. Future studies aiming for a functional cure of CHB should consider the induction of B as well as T cell immunity. Our data demonstrate that endogenous HBsAg-specific B cell responses can persist in the blood and liver of patients with CHB and start to identify targets for reconstitution of their multipronged antiviral efficacy.

## Methods

### Samples.

Acute HBV infection was diagnosed on the basis of new HBsAg positivity or recent exposure and serological evolution; samples were considered from the acute phase when HBV DNA was still detectable and ALT was greater than 40 IU/l; samples were designated as resolved phase when HBV DNA and HBsAg were undetectable and ALT was normalized. All donors with CHB were stratified for disease stage (according to cutoffs listed in figure legends) by serum HBsAg (IU/ml, Abbott Architect Quantitate HBsAg) and HBeAg status, repeated measurements of viral load (IU/ml, determined by real-time PCR), and degree of liver inflammation by serum ALT. Participants were anti–HCV and anti–HIV Ab negative unless otherwise stated; 6 controls with HCV were included, diagnosed by HCV seropositivity with RNA confirmation. All patients studied were treatment naive at the time of sampling. HC were either HBV unexposed, vaccinated with ENGERIX-B (GSK) during the study, or had anti-HBs Abs of more than 100 IU/ml following prior vaccination. HC ranged between 21 and 89 years of age; CHB patients ranged between 23 and 71 years of age.

### Preparation of PMBC and intrahepatic lymphocytes.

PBMCs were isolated from heparinized blood by density centrifugation using Ficoll-Hypaque Plus (GE Healthcare). PBMCs were either used immediately or frozen in FBS (Life Technologies) supplemented with 10% DMS0 (Sigma-Aldrich). Intrahepatic lymphocytes (IHL) from CHB liver biopsies were isolated by mechanical disruption without further processing (debris removed by filtration through 70 μM cell strainers; BD Biosciences). To isolate IHL from perfusate samples, liquid was first concentrated by centrifugation. Concentrated cells were resuspended in RPMI 1640 (Life Technologies, Thermo Fisher Scientific) and IHL isolated by density centrifugation (400 *g*) using Ficoll-Hypaque Plus. For larger explants/resections, tissue was cut into small pieces and incubated at 37°C for 30 minutes in 0.01% collagenase IV (Life Technologies) and 0.001% DNaseI (Sigma-Aldrich). Mechanical digestion was performed using a GentleMACS (Miltenyi Biotec) and debris removed by filtration. Parenchymal cells were removed by centrifugation (400 *g*) on a 30% Percoll gradient (GE Healthcare) and IHLs then isolated by density centrifugation (400 *g*) using Ficoll-Hypaque Plus.

### Flow cytometry for B cell subset phenotype and function.

Multiparametric flow cytometry was used for ex vivo analysis of B cells. For subset gating, combinations of the following mAbs were used: CD19-BV786, CD27-BUV395, CD20–Alexa Fluor 700, CD10-BV605, CD3-BV711, CD21-BV421, and CD45-BUV05. Full details of mAbs used, including those used for phenotypic characterization, are given in Supplemental Table 1.

Cells were stained with Fixable LIVE/DEAD Dye (Life Technologies, Thermo Fisher Scientific) before incubation with saturating concentrations of surface mAbs diluted in 50% Brilliant Violet Buffer (BD Biosciences) and 50% PBS for 30 minutes at 4°C. In all instances, cells were stained in the presence of FcR-blocking reagent (Miltenyi Biotec). Cells were fixed and permeabilized for further functional assessment with either Cytofix/Cytoperm (BD Biosciences) or FoxP3 Buffer Set (BD Biosciences) according to the manufacturer’s instructions for intracellular or intranculear staining respectively. Saturating concentrations of mAbs for 30 minutes at 4°C were diluted in 0.1% saponin (Sigma-Aldrich) for the detection of intracellular proteins or in 1× PBS for the detection of intranuclear proteins. All samples were acquired on a Fortessa-X20 (BD Biosciences) and analyzed using FlowJo (Tree Star).

### Identification of HBsAg-specific B cells.

For identification of HBsAg-specific B cells, 1 × 10^6^ cells were stained with an FITC-conjugated recombinant HBsAg, ayw strain (Roche). All staining was performed in parallel with mAb staining, as above, by incubating cells for 30 minutes at 4°C. Stringent gating criteria were applied with doublet, dead, and CD19-negative cell exclusion to minimize nonspecific binding contamination. Cells stained with an identical panel minus HBsAg bait (fluorescence minus one [FMO]) were used to control for nonspecific binding, with corresponding FMO frequencies subtracted. The phenotype of HBsAg-specific B cells was not analyzed in instances in which there were fewer than 50 HBsAg bait^+^ cells recorded.

### B cell stimulation.

To assess cytokine production, 2 × 10^5^ PBMCs were stimulated with either 10 μg/ml F(ab′)_2_ IgM- and IgG-specific Abs (Southern Biotech and Jackson ImmunoResearch, respectively) in combination with 0.5 μg/ml MEGACD40L (Enzo Life Sciences) or 1 μg/ml R848 (TLR7/8 agonist; Invivogen) diluted in complete RPMI (cRPMI: RPMI 1640 medium containing 10% FBS, 100 U/ml penicillin/streptomycin, 1× nonessential amino acids, 1× essential amino acids, and β-mercaptoethanol; all Life Technologies). Cells were stimulated for 16 to 18 hours in the presence of 10 μg/ml anti–PD-1 mAb or an isotype control (LEAF purified; clone EH12.2H7; BioLegend). All stimulations were performed in the presence of 1 μg/ml Brefaldin A (Sigma-Aldrich) at 37°C. Cytokine responses were analyzed by intracellular cytokine staining for IL-6 and TNF-α along with cell-surface staining to identify MBC populations.

For analysis of apoptosis, 2 × 10^5^ B cells were purified by magnetic separation according to the manufacturer’s instructions (B Cell Enrichment Kit, STEMCELL Technologies) and stimulated for 7 days with 1 μg/ml F(ab′)_2_ IgM- and IgG-specific Abs plus 500 ng/ml MEGACD40L. Cells were then stained for B cell phenotype, followed by incubation with annexin V–PerCP/Cy5.5 in the presence of annexin V–binding buffer according to the manufacturer’s instructions (BioLegend).

### Phosphoflow.

PBMCs were surface stained as above to identify MBC subsets. Cells were washed prior to stimulation with 20 μg/ml F(ab′)_2_ IgM-, IgA-, and IgG-specific Abs (Jackson ImmunoResearch) for 30 seconds. Cells were immediately fixed in prewarmed Cytofix (BD Biosciences) and left at 37°C for 10 minutes. Cells were then pelleted and resuspended in Phosflow Buffer (BD Biosciences) for 30 minutes at –20°C. Cells were stained for phosphorylated BLNK (rabbit monoclonal; Cell Signaling Technologies; catalog 3601S) for 30 minutes at 4°C. Ab was then washed off before incubation with PE secondary (Life Technologies) for a further 30 minutes at 4°C. Cells were washed and analyzed on Fortessa-X20 (BD Biosciences).

### Calcium flux.

B cells were separated through magnetic separation according to the manufacturer’s instructions (B Cell Enrichment Kit) and stained for B cell memory phenotype, as before. Cells were washed in PBS and incubated with 1 μM Fluo-4 AM Dye (Thermo Fisher) for 30 minutes at 37°C. Fluo-4 AM was diluted in RPMI containing 250 mM probenecid (Thermo Fisher). Cells were then washed in PBS and rested in the dark at room temperature (RT) for 20 minutes before analysis. 5 × 10^5^ Cells were analyzed for 20 seconds on a FORTESSA X20 (BD Biosciences) to establish baseline prior to stimulation with anti-IgM/IgG/IgA (50 μg/ml) or ionomycin (1 μg/ml), after which they were analyzed for a further 150 seconds.

### Differentiation of plasma cells.

PBMCs were stained as above and sorted into atMBC and cMBC populations using a FACSAria (BD Biosciences). Stringent criteria were applied to identify lymphocytes, based on forward scatter (FSC) versus side scatter (SSC), and to remove doublet contamination. CD10^+^ immature B cells were excluded. Between 5 × 10^4^ and 2 × 10^5^ atMBC were purified using this method; in all cases, a matched number of cMBC were sorted and stimulated as below.

Cells were first activated for 4 days in a 96-well plate using CpG-B (50 ng/ml; TLR9 agonist; ODN-2009; Invivogen) and HBsAg (Roche; ayw, 10 μg/ml) in combination with IL-2 (50 U/ml), IL-10 (50 ng/ml), IL-15 (10 ng/ml), and IL-21 (100 ng/ml), diluted in DMEM (plus 10% FBS, 1% penicillin/streptomycin, and 10% nonessential amino acids). Cells were then switched into DMEM containing IL-2, IL-10, IL-15 (concentrations as above), IL-6 (50 ng/ml), and INF-α (500 U/ml) for a further 3 days to promote their differentiation into Ab-secreting cells. After culture, cells were stained for plasma cell differentiation using BUV805-CD45, BV786-CD19, PE-Cy7-IgD, BUV395-CD27, PE-dazzle-CD38, AF700-CD20, BV711-CD3, and APC-CD138 and analyzed on a Fortessa X20.

### ELISpot.

ELISpot plates (multiscreen HTS-IP, 0.45 μm, Merck Millipore) were precoated with HBsAg (2 μg/ml) or anti-human IgG/A/M (1 μg/ml) for detection of total Ab-secreting cells and blocked with RPMI with 1% milk. For ELISpot analysis of HBsAg-specific MBC, B cells were purified from frozen PBMC samples using magnetic separation (B Cell Enrichment Kit). B cells were activated and stimulated as above. 2 × 10^5^ Cells were added to the plate and incubated at 37°C in 5% CO_2_ for 18 hours. Cells were then washed 3 times with PBS–Tween 20 (0.05%), followed by PBS. Goat anti-human IgG-horseradish peroxidase (Jackson ImmunoResearch Laboratories; diluted 1/800 in PBS–10% FBS) was added and incubated for 4 hours at RT in the dark. Cells were again washed 3 times with PBS–Tween 20 (0.05%) and 3 times with PBS, then developed with AEC substrate (BD Biosciences), according to the manufacturer’s instructions. ELISpot plates were then washed with ddH_2_0 before analysis using virusSPOT (Autoimmun Diagnostika). All assays were performed in triplicate.

### Detection of anti-HBs.

For detection of anti-HBs, HBsAg-specific B cells were sorted and stimulated for 7 days as above. Number of cells isolated ranged from between 3 × 10^3^ and 2 × 10^4^. Culture supernatants were sent for anti-HBs quantification (Health Services Laboratory, University College London Hospitals NHS Foundation Trust, Abbott Architect anti-HBs). Samples were also tested in parallel in-house using the anti-HBs CLIA Kit (Autobio) per the manufacturer’s instructions.

### tSNE.

tSNE analysis was performed on concatenated flow cytometry data from 10 patients with CHB and 10 HC using default parameters (iterations, 1,000; perplexity, 20; and θ, 0.5). tSNE was applied to expression data for CD27, CD21, PD-1, FcRL5, CD24, CD38, IgM, and IgD for all live CD45^+^CD19^+^CD3^–^CD20^–^CD10^–^ events.

### Statistics.

Statistical analyses were performed in Prism (GraphPad) as indicated in legends: Kruskal-Wallis test (ANOVA) with Dunn’s post hoc test for pairwise multiple comparisons; Spearman’s rank correlation; Mann-Whitney unpaired *t* test; and Wilcoxon’s paired *t* test. All tests were carried out as 2-tailed tests. Significance was defined as *P* < 0.05.

### Study approval.

This study was approved by the local ethical boards of London-Brent (REC number 16/LO/1699) and Brighton and Sussex (REC number 11/LO/0421). Each participant gave written informed consent. Storage of samples complied with the requirements of the Data Protection Act of 1998 and the Human Tissue Act of 2004. Resected liver samples from healthy margins of colorectal metastatic tumor resections were obtained through the Tissue Access for Patient Benefit (TAPb) scheme at The Royal Free Hospital (approved by UCL–Royal Free Hospital BioBank Ethical Review Committee, 11/WA/0077). Perfusion liquid was obtained from healthy livers prior to solid-organ transplantation (REC number 11/H0720/4). HBV-infected liver tissue was obtained from liver biopsies deemed surplus to diagnostic requirements in treatment-naive CHB patients attending clinics at The Royal London Hospital (approved by East London and The City REC number P/01/023; and London-Brent REC number 16/LO/1699). Five liver samples were obtained from patients with HBV-related end-stage cirrhosis undergoing resection surgery (TAPb).

## Author contributions

NP and MKM conceived the project and obtained funding. ARB, NP, and MKM designed experiments. ARB, KS, and EA generated data. ARB, LJP, LEM, LS, CM, PAB, and MKM analyzed and interpreted data. OEA, BRD, USG, and PTFK provided essential patient samples and clinical data. ARB and MKM prepared the manuscript. All other authors provided critical review of the manuscript.

## Supplementary Material

Supplemental data

## Version 1 08/09/2018

In-Press Preview

## Version 2 09/13/2018

Electronic publication

## Version 3 09/13/2018

Electronic publication

## Version 4 09/17/2018

Electronic publication

## Version 5 10/01/2018

Print issue publication
