# Supplementary material for: Circulating and intrahepatic antiviral B cells are defective in hepatitis B
Source: J Clin Invest. 2018 Aug 9;128(10):4588–603. doi: 10.1172/JCI121960 (PMC6159997; doi:10.1172/JCI121960)
Supplement: Supplemental data [file jci-128-121960-s322.pdf]

Supplementary Figure. 1.

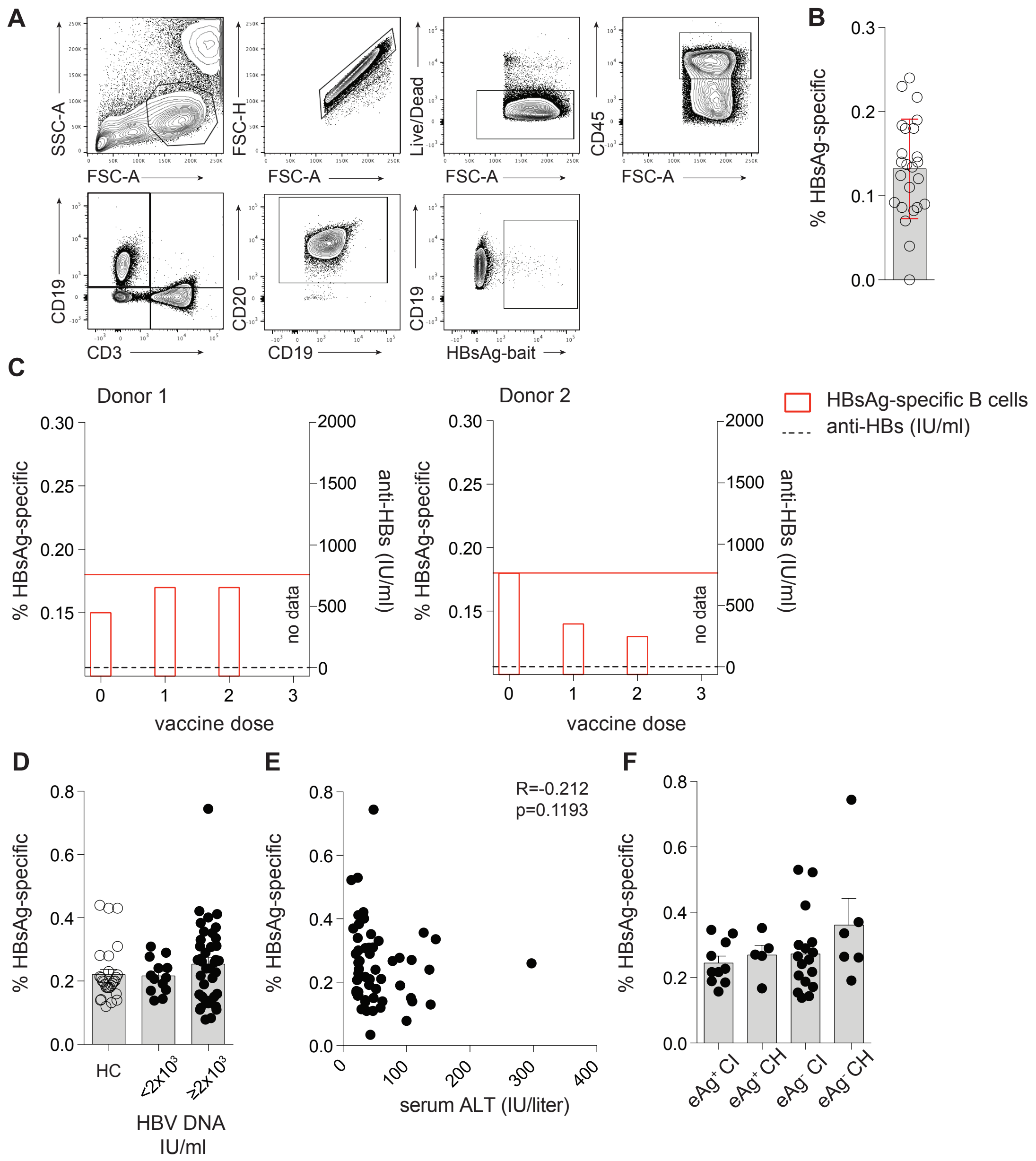

Supplementary Figure 1. Identification of HBsAg-specific B cells in patients with CHB
A. Sequential multiparametric flow cytometric gating strategy used for the identification of HBsAg-specific B cells. **B.** HBsAg-bait staining of unexposed healthy controls (n=24). Error bars show the s.d of the data used to define the background threshold for detection of HBsAg-specific B cells (set at mean + s.d. of unexposed, y=0.18% of $CD19^+CD20^+$). **C.** Frequency of HBsAg-specific B cells (red bars; % of $CD19^+CD20^+$) in two healthy donors who did not complete their course of HBV-vaccination. Red line delineates threshold level based on mean + s.d of unexposed controls (0.18). **D.** Summary plot of the frequencies of HBsAg-specific B cells stratified by HBV viral load: n=29 vac HC; n=13 with HBV DNA $<2x10^3$ IU/ml; and n=39 with HBV $\geq 2x10^3$ IU/ml. **E.** Correlative analysis of the frequency of HBsAg-specific B cells and serum ALT (IU/l; n=84). **F.** Summary plot of the frequencies of HBsAg-specific B cells stratified by CHB disease phase: n=10 'HBeAg$^+$ chronic infection' (eAg$^+$ CI, HBeAg$^+$, HBV viral load > 10^7 IU/ml, serum ALT < 40 IU/liter); n=5 'HBeAg$^+$ chronic hepatitis' (eAg$^+$ CH, HBeAg$^+$, HBV viral load > 5 x 10^5 IU/ml, serum ALT > 60 IU/liter); n=17 'HBeAg$^-$ chronic infection' (eAg$^-$ CI; HBeAg$^-$, HBV viral load < 2000 IU/ml, serum ALT < 40 IU/liter); and n=6 'HBeAg$^-$ chronic hepatitis' (eAg$^-$ CH, HBeAg$^-$, HBV viral load > 5 x 10^5 IU/ml, serum ALT > 60 IU/liter). Error bars indicate means ± SEM; *, P < 0.05; **, P < 0.01; ***, P < 0.001; ****, P < 0.0001; p-values were determined via Kruskal-Wallis test (ANOVA) with a Dunn's post hoc test for pairwise multiple comparisons (d and f); and Spearman's Rank correlation (e).

Supplementary Figure. 2.

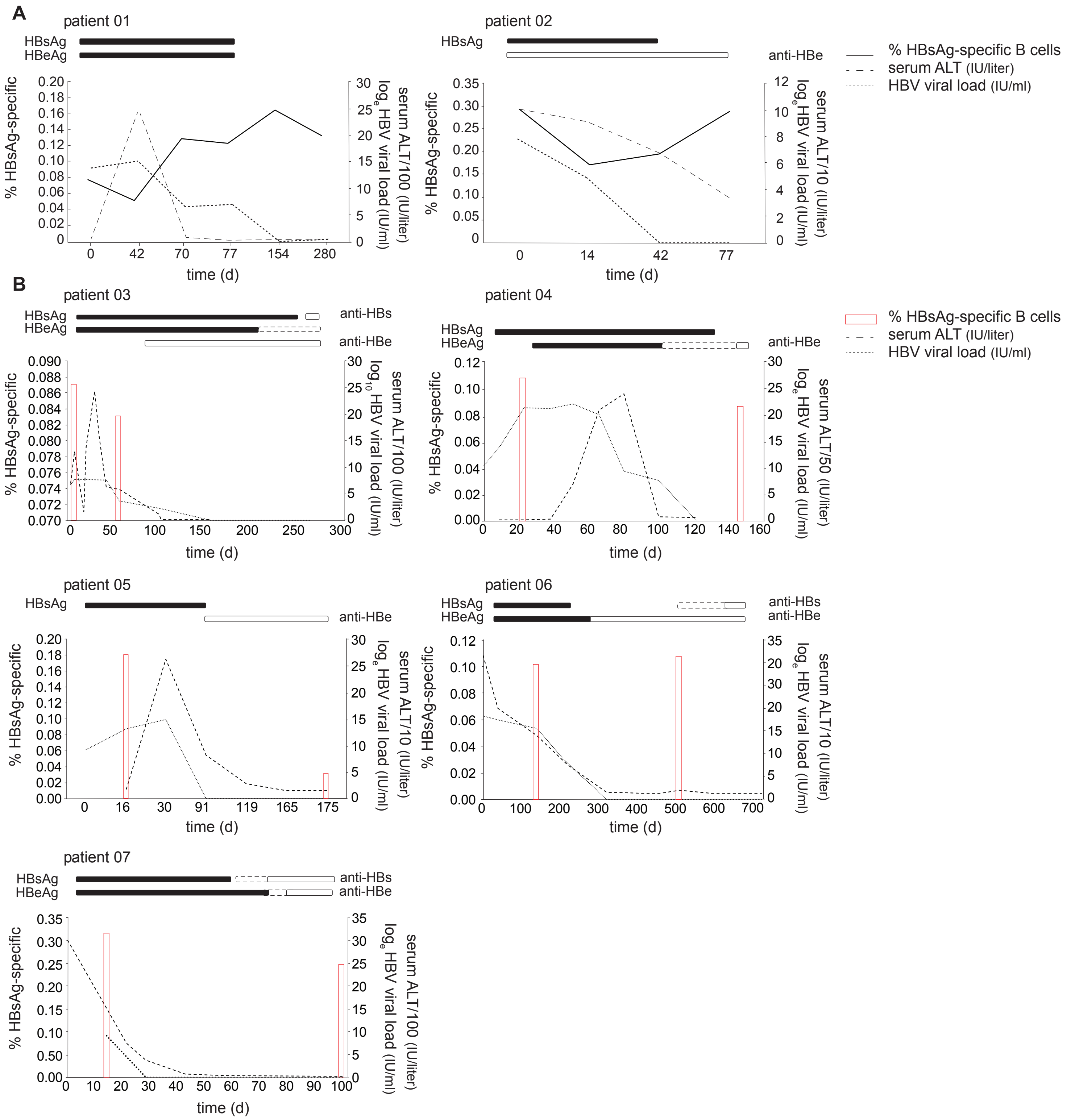

Supplementary Figure 2. Clinical sampling of HBV-acute and -resolving patients
A. Longitudinal analysis of HBsAsg-specific B (% of CD20^{+}CD19^{+}) cells during acute-resolving infection. Percentage of HBsAg-specific B cells (black line) are plotted in relation to viral load (dotted line; IU/ml), serum ALT (dashed line; IU/liter) and serological status as indicated by the bars. **B.** Clinical data from longitudinal sampling of 5 acute-resolving patients, showing viral load (dotted line; IU/ml), serum ALT (dashed line; IU/liter), and serological data indicated by the bars (solid bars delineate presence of HBV-viral antigen; clear bars delineate presence of HBV-viral IgG; dashed bars used where data is not known). Frequency of HBsAg-specific B cells measured at cross-sectional timepoints during infection is indicated by the red bars.

Supplementary Figure. 3.

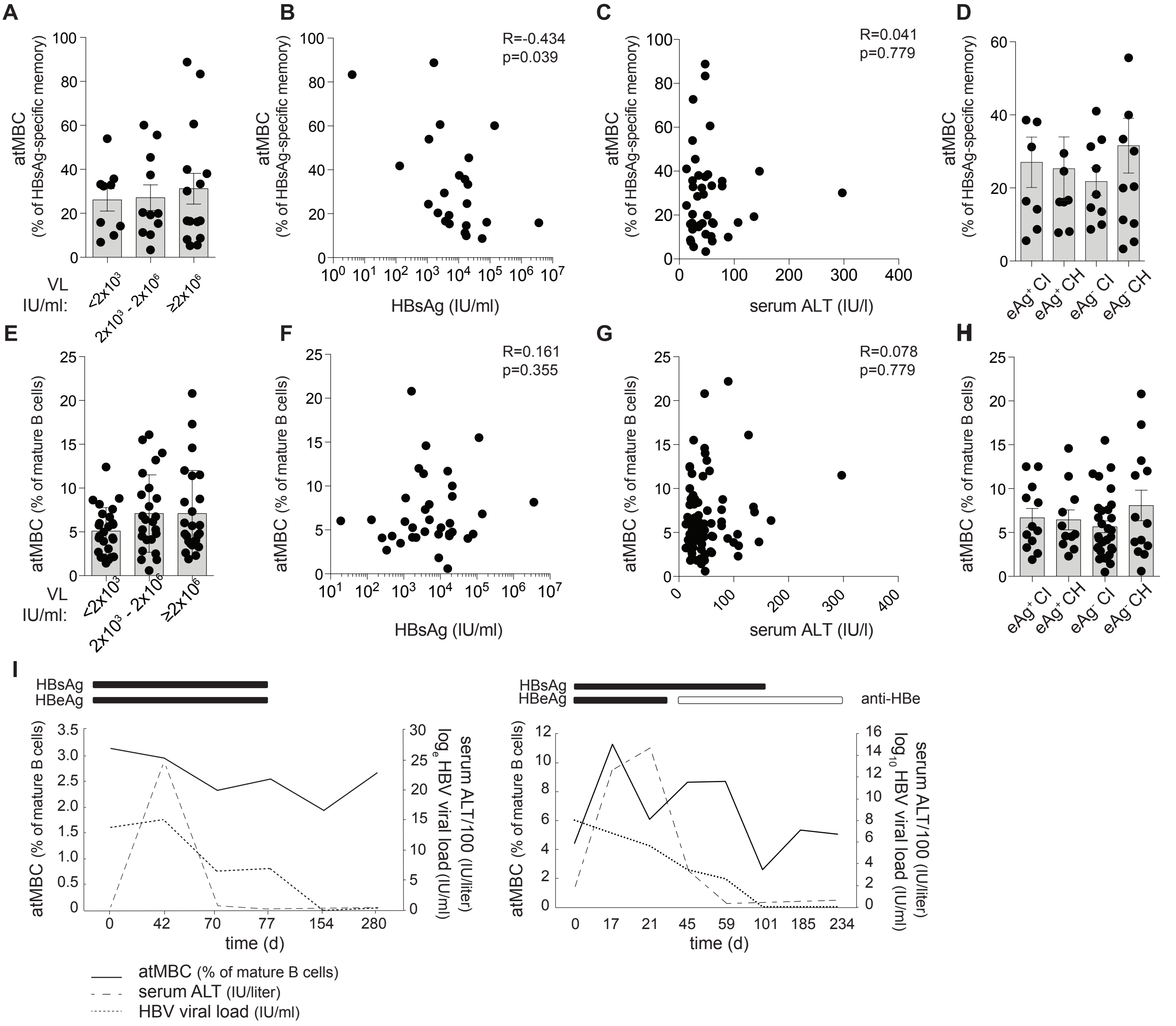

Supplementary Figure 3. Associations between clinical parameters and memory B cell subsets

A. Frequencies of atMBC as a proportion of HBsAg-specific memory B cells, stratified by viral load: n=9 with HBV DNA <2x10^3 IU/ml; n=11 with HBV DNA 2x10^3 - 2x10^6 IU/ml; and n=15 with HBV ≥ 2x10^6 IU/ml; and **B.** correlated with HBsAg (IU/ml; n=38) or **C.** serum ALT (n=42). **D.** Frequency of HBsAg-specific atMBC stratified by disease phase: n=8 'HBeAg$^+$ chronic infection' (eAg$^+$ CI; HBeAg$^+$, HBV viral load > 10^7 IU/ml, serum ALT < 40 IU/liter); n=5 'HBeAg$^+$ chronic hepatitis' (eAg$^+$ CH, HBeAg$^+$, HBV viral load > 5 x 10^5 IU/ml, serum ALT > 60 IU/liter); n=10 'HBeAg$^-$ chronic infection' (eAg$^-$ CI; HBeAg$^-$, HBV viral load < 2000 IU/ml, serum ALT < 40 IU/liter); and n= 6 'HBeAg$^-$ chronic hepatitis' (eAg$^-$ CH, HBeAg$^-$, HBV viral load > 5 x 10^5 IU/ml, serum ALT > 60 IU/liter). **E.** Frequency of global atMBC stratified by viral load: n=26 with HBV DNA <2x10^3 IU/ml; n=24 with HBV DNA <2x10^3 - 2x10^6 IU/ml; and n=25 with HBV ≥ 2x10^6 IU/ml; and **F.** correlated with HBsAg (IU/ml; n = 57) or **G.** serum ALT (n=84). **H.** Frequency of global atMBC stratified by disease phase as defined above: n=12 'eAg$^+$ CI'; n=11 'eAg$^+$ CH'; n=30 'eAg$^-$ CI'; and n=13 'eAg$^-$ CH'. **I.** Longitudinal analysis of atMBC during acute-resolving infection. Frequencies plotted in relation to viral load (dashed line; IU/ml), serum ALT (dotted line; IU/liter) and serological status, indicated by the bars. Data for serum ALT and HBV viral load is also shown in figure 1G and supplementary figure 2. Error bars indicate mean ± SEM; *, P < 0.05; **, P < 0.01; ***, P < 0.001; ****, P < 0.0001; p-values were determined by Kruskal-Wallis test (ANOVA) with a Dunn's post hoc test for pairwise multiple comparisons (a, d, e and h); and Spearman's Rank correlation (b, c, f and g).

Supplementary Figure. 4.

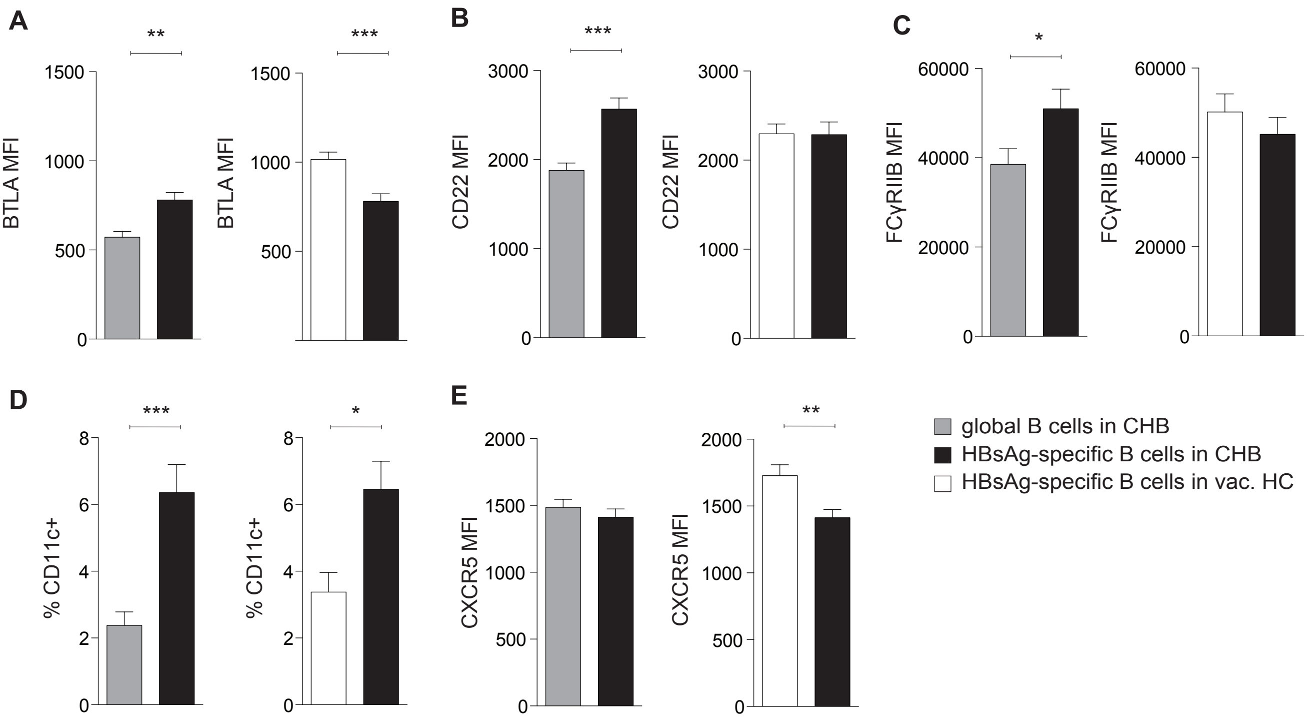

Supplementary Figure 4. Phenotypic analysis of HBsAg-specific B cells

A-E. Paired analysis of marker expression on HBsAg-specific B cells (black) compared to global B cells (grey) from within the same patient with CHB, and comparison of HBsAg-specific B cells in patients with CHB and vaccinated healthy controls (white; vac HC). Expression levels of **A.** BTLA (MFI; n=9 patients with CHB; n=13 vac HC), **B.** CD22 (MFI; n=15 patients with CHB; n=18 vac HC), **C.** FcyRIIB (MFI; n=14 patients with CHB; n=18 vac HC), **D.** CD11c (%; n=33 patients with CHB; n=16 vac HC), and **E.** CXCR5 (MFI; n=33 patients with CHB; n=17 vac HC). Error bars indicate mean ± SEM; *, $P < 0.05$; **, $P < 0.01$; ***, $P < 0.001$; ****, $P < 0.0001$; p-values determined by Wilcoxon paired t test (for paired data) and Mann-Whitney t test (for unpaired data).

Supplementary Figure. 5.

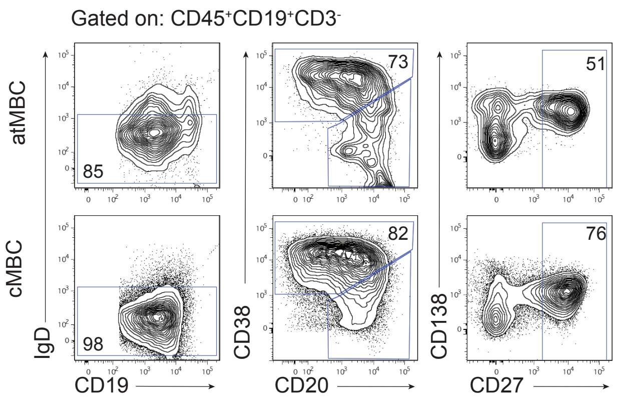

Supplementary Figure 5. Plasma cell differentiation of memory B cell subsets
Sequential gating strategy and representative staining showing the proportion of cells that acquire a plasma cell phenotype in atypical compared to classical memory subsets. atMBC were FACS sorted from healthy control blood (n=7) and differentiated into plasma cells, alongside a matched number of cMBC. Cells were analysed by flow cytometry, with plasma cells defined as $CD45^{+}CD19^{+}CD3^{-}IgD^{-}CD38^{hi}CD20^{-}CD27^{+}CD138^{+}$.

Supplementary Table 1: Monoclonal antibody details

Antigen	Fluorochrome	Clone	Manufacturer	Catalog number
**Phenotype**				
CD45	BUV805	HI30	BD	564914
CD19	BV786	SJ25C1	BD	563325
CD19	eFluro450	HIB19	eBioscience	48-0199-42
CD20	Alexa-Fluor700	2H7	BD	560631
CD3	BV711	OKT3	BioLegend	317328
CD3	BV510	OKT3	BioLegend	317332
CD10	BV605	Ber-ACT8	BioLegend	350218
CD21	BV421	B-Ly4	BD	562996
CD21	APC	B-Ly4	BD	561767
CD27	BUV395	L128	BD	561767
IgM	APC/Cy7	MHM-88	BD	314520
IgD	PE/Cy7	IA6-2	BD	561314
IgD	PerCP-Cy5.5	IA6-2	BioLegend	348208
PD-1	PE	EH12.2H7	BioLegend	329906
FcRL5	APC	509f6	BioLegend	340306
CD32b	PE	FUN-2	BioLegend	303205
BTLA	APC/Cy7	MIH26	BioLegend	344517
CD22	PE/Cy7	HIB22	BioLegend	302514
CD80	BV510	L307.4	BD	563084
CD11c	BV711	3.9	BioLegend	301630
CXCR5	APC/Cy7	J252D4	BioLegend	356925
CXCR3	PerCP-Cy5.5	IC6/CXCR3	BD	560832
CD24	PE/Cy7	ML5	BioLegend	311120
CD38	PE-dazzle	HIT2	BioLegend	303532
CD138	APC	DL-101	BioLegend	352307
T-bet	EFluor666	EBio4B10	eBioscience	50-5825-82
**Function**				
TNF-α	FITC	MAb11	BD	554512
Interleukin-6	APC	MQ2-13A3	BioLegend	501112
Annexin-V	PerCP-Cy5.5		BioLegend	B225753
